# Metabarcoding analysis identifies high diversity of harmful algal bloom species in the coastal waters of the Beibu Gulf

**DOI:** 10.1002/ece3.10127

**Published:** 2023-05-22

**Authors:** Liyan He, Zhiming Yu, Xin Xu, Jianan Zhu, Yongquan Yuan, Xihua Cao, Xiuxian Song

**Affiliations:** ^1^ CAS Key Laboratory of Marine Ecology and Environmental Sciences Institute of Oceanology, Chinese Academy of Sciences Qingdao China; ^2^ Functional Laboratory of Marine Ecology and Environmental Science Laoshan Laboratory Qingdao China; ^3^ Center for Ocean Mega‐Science Chinese Academy of Sciences Qingdao China; ^4^ University of Chinese Academy of Sciences Beijing China

**Keywords:** Beibu Gulf, diversity, harmful algal species, long‐read, metabarcoding, short‐read

## Abstract

Harmful algal blooms (HABs) have occurred more frequently in recent years. In this study, to investigate their potential impact in the Beibu Gulf, short‐read and long‐read metabarcoding analyses were combined for annual marine phytoplankton community and HAB species identification. Short‐read metabarcoding showed a high level of phytoplankton biodiversity in this area, with Dinophyceae dominating, especially Gymnodiniales. Multiple small phytoplankton, including Prymnesiophyceae and Prasinophyceae, were also identified, which complements the previous lack of identifying small phytoplankton and those unstable after fixation. Of the top 20 phytoplankton genera identified, 15 were HAB‐forming genera, which accounted for 47.3%–71.5% of the relative abundance of phytoplankton. Based on long‐read metabarcoding, a total of 147 OTUs (PID > 97%) belonging to phytoplankton were identified at the species level, including 118 species. Among them, 37 species belonged to HAB‐forming species, and 98 species were reported for the first time in the Beibu Gulf. Contrasting the two metabarcoding approaches at the class level, they both showed a predominance of Dinophyceae, and both included high abundances of Bacillariophyceae, Prasinophyceae, and Prymnesiophyceae, but the relative contents of the classes varied. Notably, the results of the two metabarcoding approaches were quite different below the genus level. The high abundance and diversity of HAB species were probably due to their special life history and multiple nutritional modes. Annual HAB species variation revealed in this study provided a basis for evaluating their potential impact on aquaculture and even nuclear power plant safety in the Beibu Gulf.

## Graphical abstract

This study firstly combined both short‐read and long‐read metabarcoding analyses for annual marine phytoplankton community and harmful algal bloom species identification in the Beibu Gulf. Based on the high richness from short‐read and the high accuracy of species identification from long‐read, HAB species with high diversity and abundance were revealed. The metabarcoding analyses also make up the previous light microscopic method in identifying specific phytoplankton species, such as those with small‐size, those unstable after fixation, and those lack obvious morphological differences in the same genus.

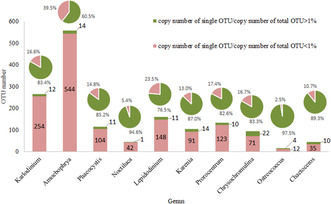

## 1 INTRODUCTION

Harmful algal blooms (HABs) are ecological disasters mainly caused by the massive growth of phytoplankton and other species including cyanobacteria, leading to a massive release of toxins, water discoloration or generation of anoxic conditions (Anderson et al., 2012; Hallegraeff et al., 2003). In recent years, ocean warming and eutrophication have increased with intensified human activities, which have triggered more HABs and led to important socio‐ecologic‐economic impacts. At present, approximately 300 species form HABs are deleterious to aquatic ecosystems (Graneli & Turner, 2006). The impact of HABs is expanding, and they have become common marine ecological disasters that limit the development of offshore economies, threaten human food safety, destroy marine ecosystems, and even threaten the safety of nuclear power plants (Anderson, 2003; Gu et al., 2022; Yu et al., 2017).

The Beibu Gulf, a semiclosed bay between China and Vietnam, is located in the northwest region of the South China Sea and is also an important fishing ground and aquaculture area in China (Crook et al., 2003). Before 2005, HABs recorded in the sea area were mainly caused by cyanobacteria such as *Trichodesmium erythraeum*, *Trichodesmium hildebrandtii*, *Microcystis aeruginosa*, and *Microcystis aquatica*, and the outbreak of these HABs affected the health of the marine ecological environment (Jiang, 2019; Luo et al., 2016). While since 2005, the types of HABs have been diversifying, including *Noctiluca* sp., *Phaeocystis globosa*, and *Leptocylindrus danicus*, which damage the marine ecological environment and introduce new hazards (Jiang, 2019). For example, in April 2011, a *Noctiluca* sp. bloom in Qinzhou Bay led to a large number of fish mortalities (Luo et al., 2016). In 2015, the outbreak of *P*. *globosa* bloom blocked the water‐cooling system in the Fangchenggang nuclear power plant, and it was the first case in which HAB affected the safety of a coastal nuclear power station in the world (Yu et al., 2017). These newly emerging HABs have caused various types of disasters based on their different biological characteristics. To evaluate the kind of risk HABs could generate in the Beibu Gulf, it is necessary to identify the phytoplankton community, especially HAB species, and their dynamic changes in this sea area.

Morphological taxonomy based on microscopic observation is a traditional approach for identification of phytoplankton species and is suitable for distinguishing species with visible morphological differences. In recent years, the metabarcoding approach has been increasingly applied for identification of phytoplankton species, with the advantage of identifying both small‐sized cells and those difficult to preserve (Chen et al., 2022; Zimmermann et al., 2015). For the metabarcoding approach, the short‐reads of the small subunit ribosomal ribonucleic acid (SSU rRNA) are most frequently used, such as the V4 region (Cheung et al., 2010; Ribeiro et al., 2018) and the V9 region (de Vargas et al., 2015; dos Santos et al., 2017). To evaluate HAB species, metabarcoding approach has been widely applied in the coastal waters of China, including the Bohai Sea (Xu et al., 2017), Jiaozhou Bay (Liu, Gibson, et al., 2020), East China Sea (Chen et al., 2021), Changjiang estuary (Cui et al., 2021), and South China Sea (Wang et al., 2022). However, due to the restricted sequence length, short‐read metabarcoding approaches using the V4 or V9 region can usually identify phytoplankton at the genus level but not the species level, especially in some closely related species. As the sensitivity and accuracy of species identification increases with increasing sequence read length (Yarza et al., 2014), compared with short‐read metabarcoding, long‐read metabarcoding can greatly increase the resolution of species‐level identifications. Thus, based on the high resolution of long‐read metabarcoding, the possible sources of new emerging HAB species could be clarified. With the development of Nanopore Sequencing, obtaining full‐length sequences of SSU rRNA from environmental samples has become possible. At present, Single molecule real‐time (SMRT) technology based on the PacBio platform has been widely used to elucidate prokaryotic community structures (Pootakham et al., 2017; Wagner et al., 2016).

The outbreak of algal blooms represented by *P*. *globosa* poses new threats in the Beibu Gulf (Yu & Chen, 2019). Therefore, it is necessary to identify other HAB species to evaluate the potential impact of these HAB species on aquaculture and even nuclear power plant safety. In this study, based on eight surveys in the coastal waters of the Beibu Gulf in 2016–2017, the phytoplankton community and HAB species were systematically investigated by employing both short‐read and long‐read metabarcoding approaches, to provide a basis for elucidating the potential impact of HAB species in the Beibu Gulf.

## 2 MATERIALS AND METHODS

### 2.1 Sampling collection in the Beibu Gulf

**FIGURE 1:**
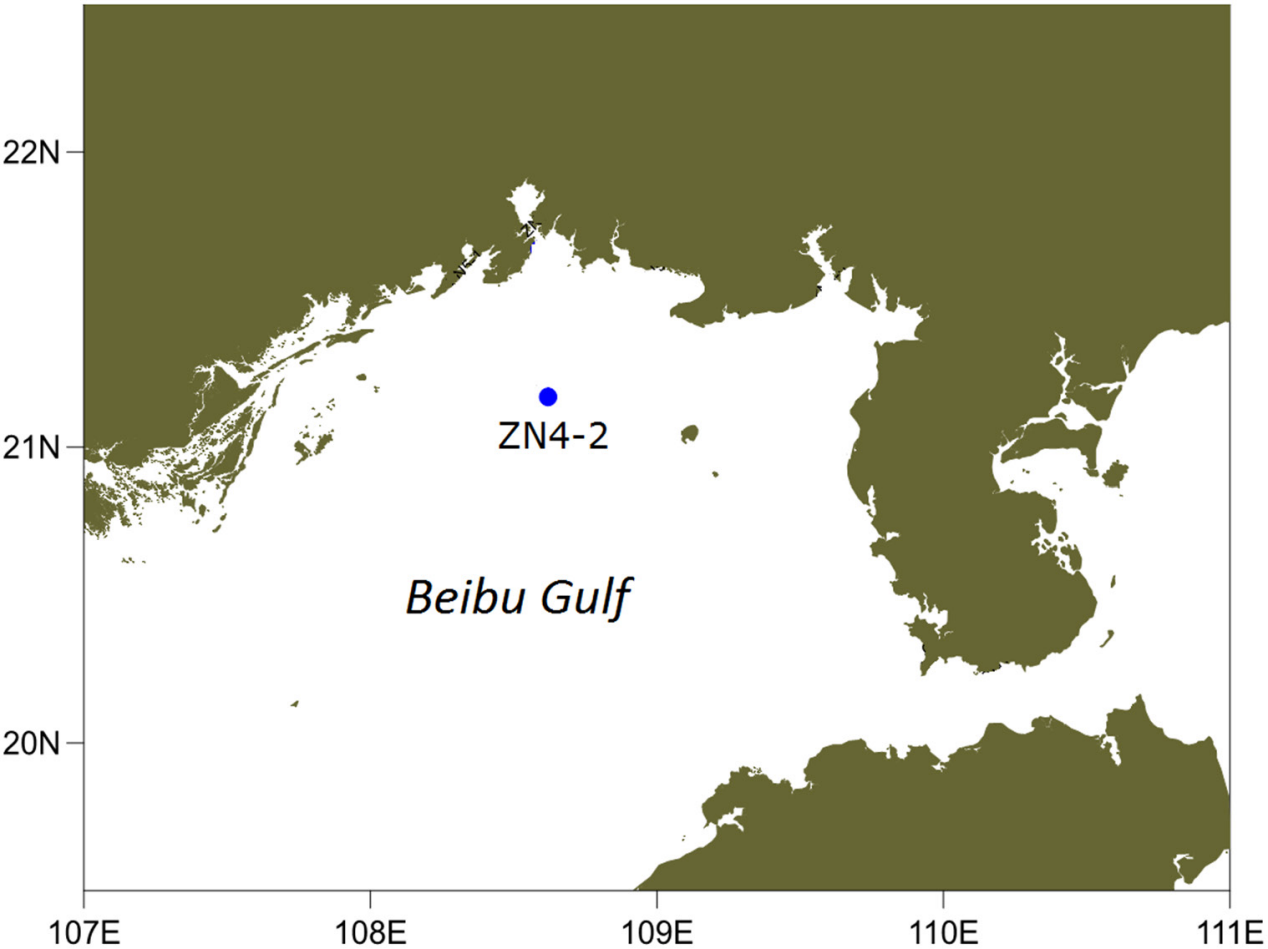
Sampling station in Beibu Gulf.

In this study, samples were collected at ZN4‐2 (108°37′12″E, 21°10′12″N), a fixed offshore station with water depth of approximately 20 m in the Beibu Gulf (Figure 1). During 2016–2017, a total of 8 investigations (2016/09, 2016/11, 2016/12, 2017/01, 2017/02, 2017/03, 2017/06, and 2017/08) were carried out, spanning the four seasons of the year. In the different periods, surface waters were first collected in plastic buckets through a vacuum pump with the inlet side filtered by a 200 μm net to eliminate interference from zooplankton. Then, phytoplankton samples were further collected on 0.68 μm GF/F membranes with a diameter of 15 cm by a vacuum pump. As the phytoplankton cell density varied in different periods, an unified volume of water means different phytoplankton biomass. To acquire relatively consistent biomass enough for DNA extraction, we collected a variety of volumes by filtering within the same time. The filtered water volume for each sample ranged from 11 to 30 L depending on the phytoplankton biomass to acquire enough cells for DNA extraction (Table 1). The sample membranes were folded and placed in 15 mL centrifuge tubes, which were immediately stored in liquid nitrogen. The above processes were all performed in the research vessel. Finally, organisms between 0.68 and 200 μm were collected for further investigation.

**TABLE 1:** Filtrated water volumes for each sample.

Sample time	2016/09	2016/11	2016/12	2017/01	2017/02	2017/03	2017/06	2017/08
Volume (L)	25	30	30	11	18	20	22	25

### 2.2 DNA extraction and fragment amplification

DNA was extracted from the samples collected on GF/F membranes (0.68 μm) according to the instructions of the Extraction Kit (MicroElute Genomic DNA Kit, Omega) and further purified. In addition, 1% agarose gel electrophoresis was used to detect the quality of the extracted DNA. For each sample, the extracted DNA amplified both the V4 region and full‐length sequence of SSU rRNA using universal primers for phytoplankton. The V4 region amplification primers were 528F forward primer (GCGGTAATTCCAGCTCCAA) and 706R reverse primer (AATCCRAGAATTTCACCTCT; Cheung et al., 2010). The full‐length amplification primers were 28F forward primer (CGA ATT CAA CCT GGT TGA TCC TGC CAG T) and 42R reverse primer (CCG GAT CCT GAT CCT TCT GCA GGT TCA CCT AC; Gu & Wang, 2007). All PCR reactions were performed with 1 μL forward primer, 1 μL reverse primer, 15 μL PCR Phusion Master Mix with GE Buffer (Biolabs) and 1 ng DNA template in a final volume of 30 μL. The reaction conditions were as follows: initial denaturation at 94°C for 1 min, followed by 30 cycles of denaturation at 94°C for 1 min, annealing at 58°C for 30 s, and elongation at 72°C for 2 min, with a final extension at 72°C for 3 min. PCR products were run on a 2% agarose gel to check amplicon lengths. For each sample, the GeneJET Gel Extraction Kit (Thermo Scientific) was employed for PCR replicate purification.

### 2.3 Sequencing analysis method

The short‐read and long‐read metabarcoding were based on the sequence of the V4 region and the full‐length sequence of SSU rRNA amplified from each sample and were further sequenced by the Illumina MiSeq platform and PacBio SMRT sequencing, respectively.

The short‐read metabarcoding used an Illumina MiSeq platform to perform paired‐end sequencing on the community DNA fragments. After sequencing to obtain the original data, we filtered the low‐quality reads (FLASH, v1.2.7; Magoc & Salzberg, 2011), assembled them, spliced the two end reads into tags, and then filtered the tags to obtain clean tags. Next, Quantitative Insights into Microbial Ecology (QIIME) v1.8.0 was used to remove the chimeric tags detected during cluster comparison (Caporaso et al., 2010). After obtaining OTUs, OTU abundance statistics were obtained based on the effective tags. The OTUs with abundance values lower than 0.001% of the total sequencing amount of all samples were removed (Bokulich et al., 2013), and the abundance matrix obtained after the removal of rare OTUs was used for the series of subsequent analyses. Sequences with a similarity of more than 97% with the representative sequences of OTUs were selected, and an OTU table was generated (Table S2). The Chao1 estimator (Chao, 1984), the ACE estimator (Chao & Yang, 1993), Shannon diversity index (Shannon, 1948), and the Simpson index (Simpson, 1949) were employed to reflect the alpha diversity of phytoplankton.

The long‐read metabarcoding was performed with PacBio SMRT sequencing software, and SMRT link v7.0 software was used to preprocess and filter the original sequencing output data to obtain circular consensus sequences (CCSs), namely, raw reads. Finally, the raw reads were filtered by length (1000–2000 bp), the primers were removed, and the chimeras were removed to obtain clean reads for subsequent analysis. To study the species composition diversity, the clean CCS of all samples were OTU clustered with 97% identity by using Mothur software (v.1.34.4; Schloss et al., 2009). During the clustering process, chimeras were removed to obtain the representative sequences of OTUs. Then, all optimized sequences were mapped to the representative sequences of OTUs. Sequences with a similarity of more than 97% with the representative sequences of OTUs were selected, and an OTU table was generated.

The taxonomic information for each OTU was annotated with RDP classifier software (Caporaso et al., 2011), and the SILVA database (Release 123) was used. To more closely study the abundance and richness of phytoplankton, for the 18S rDNA results obtained by the above two sequencing approaches, this study removed the OTUs of nonalgae in each taxonomic system, including alignment to Metazoan and unclassified data. The relative abundance of each taxon was calculated by comparing the sequence number of OTUs in this group divided by the total sequence number of algae.

### 2.4 Phytoplankton community structural comparisons and phylogenetic construction

Comparisons were carried out using the SILVA database (Quast et al., 2013), and phylogenetic relationships were constructed using MEGA 5 (Tamura et al., 2011). We downloaded the relevant sequences of SSU rRNA from the SILVA database (release 123). For each HAB species, the fraction with OTUs greater than 1% was selected for phylogenetic analysis. Phylogenetic analysis, pairwise alignment and multiple alignments of target sequences were performed by ClustalW progress, and then the sequence was intercepted at the same position for the construction of phylogenetic relationships. Evolutionary history was inferred by using the maximum likelihood method based on the Tamura‐Nei model (Tamura & Nei, 1993). Initial tree(s) for the heuristic search were obtained automatically as follows. When the number of common sites was <100 or less than one‐fourth of the total number of sites, the maximum parsimony method was used; otherwise, the BIONJ method with an MCL distance matrix was used. The tree was drawn to scale, with branch lengths measured in the number of substitutions per site. All positions containing gaps and missing data were eliminated. Evolutionary analyses were conducted in MEGA5 (Tamura et al., 2011).

### 2.5 Redundancy analysis of dominant phytoplankton genera and key environmental factors

With the advantage of generating larger amount of clean reads and resulting more phytoplankton information, short‐read metabarcoding already have sufficient taxonomy resolution at the genus level. Thus, we chose the top 10 dominant genera of phytoplankton obtained in this study by short‐read metabarcoding, combined with environmental factors, including ammonia nitrogen (${\mathrm{NH}}_{4}^{+}‐N$), nitrate nitrogen (${\mathrm{NO}}_{3}^{-}‐N$), nitrite nitrogen (${\mathrm{NO}}_{2}^{-}‐N$), phosphate (${\mathrm{PO}}_{4}^{3-}‐P$), silicate (${\mathrm{SiO}}_{3}^{2-}‐\mathrm{Si}$), total inorganic nitrogen (DIN), and total inorganic nitrogen/total inorganic phosphate (DIN/DIP) for redundancy analysis. The VEGAN package of R was used for implementation (Dixon, 2003). The relevant environmental factor data above were referenced from previously published studies (Yuan et al., 2019).

## 3 RESULTS

### 3.1 Annual variation of phytoplankton community

Based on the short‐read metabarcoding results, we obtained the biodiversity index of samples from 8 periods (Table 2). From the perspective of the Chao1 and ACE indices, the maximum values in August 2017 and September 2016 indicated that the phytoplankton community richness and diversity were the highest during these two months, while the phytoplankton community richness and diversity were the lowest in June 2017 and December 2016. The Shannon index values were the highest in September 2016 and August 2017, indicating that the phytoplankton evenness and community diversity were the highest during these two months, while the phytoplankton evenness in June 2017 and December 2016 was low, and the community diversity was the lowest. The Simpson index values were the greatest in September 2016 and August 2017, indicating that the phytoplankton evenness and community diversity were the highest during these two months, while the phytoplankton evenness and community diversity were the lowest in June 2017 and March 2017. The above four index values indicated that, overall, the diversity of the phytoplankton community was the highest in August 2017 and September 2016 and was the lowest in June 2017.

**TABLE 2:** Alpha diversity indices of phytoplankton based on the V4 region.

Sample time	Chao1	ACE	Shannon	Simpson
2016/9	1415.91	1439.99	8.10	0.9880
2016/11	707.49	703.45	6.00	0.9354
2016/12	621.65	616.62	5.55	0.9466
2017/01	1287.52	1284.94	7.11	0.9677
2017/02	797.00	797.00	6.54	0.9572
2017/03	975.69	1006.61	6.00	0.9273
2017/06	560.25	560.16	5.03	0.9062
2017/08	1417.00	1428.62	7.56	0.9756

*Note*: The first column in the table refers to the collection timepoint of samples, and the subsequent columns correspond to the calculation results for the Chao1, ACE, Shannon and Simpson diversity indices of each sample at the same sequencing depth.

**FIGURE 2:**
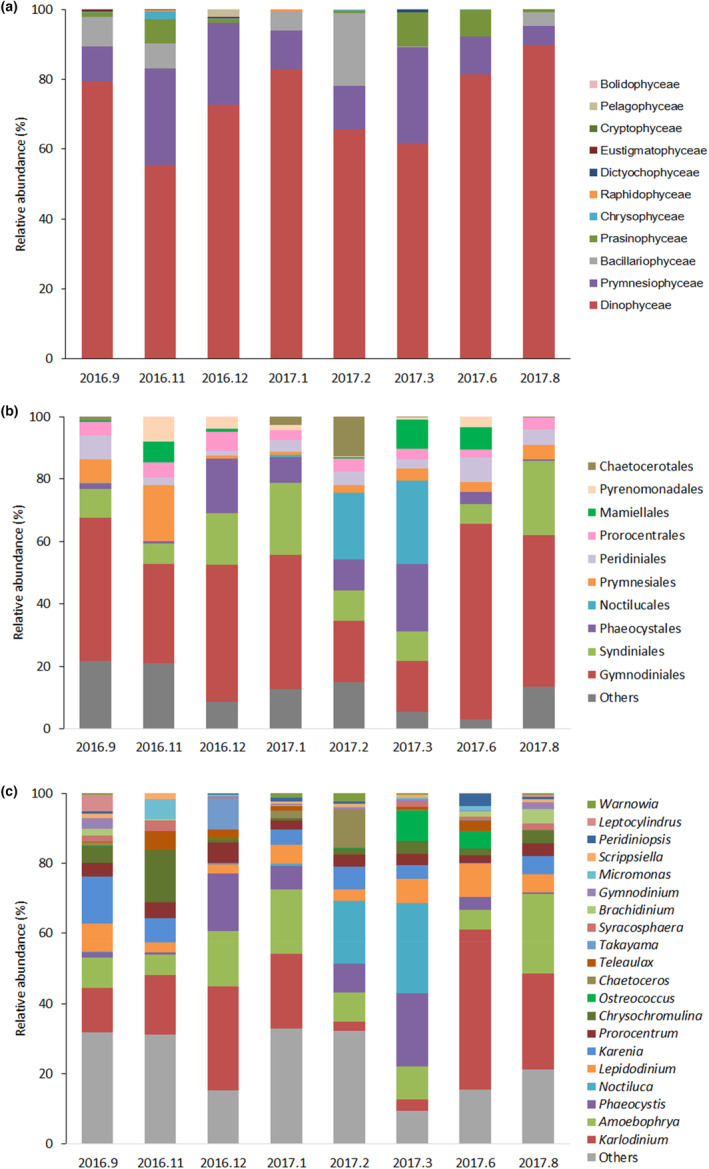
Relative abundance of phytoplankton based on the 18S rDNA V4 region. (a) class level (b) order level (c) genus level.

**FIGURE 3:**
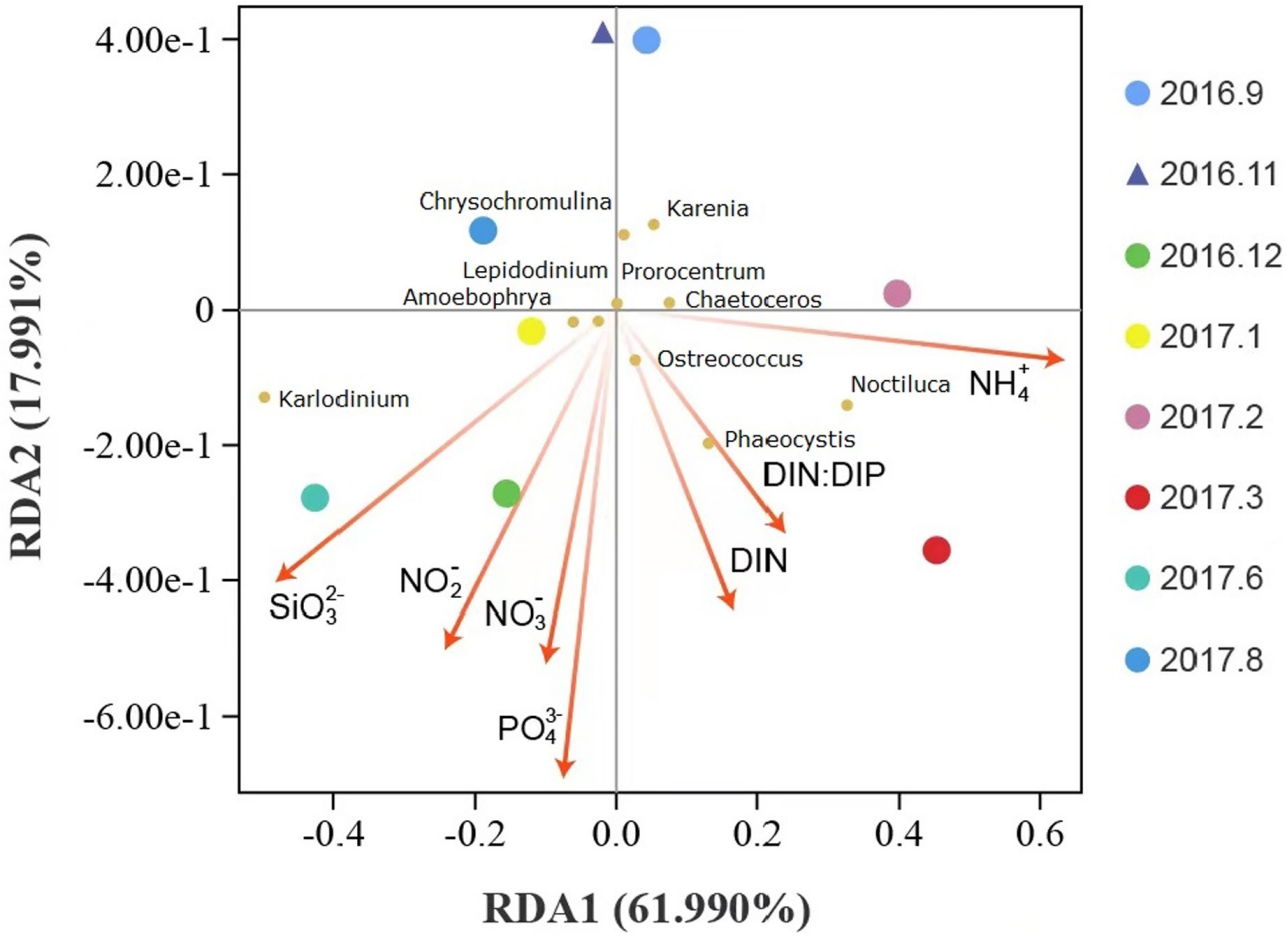
RDA between the relative abundance of the top 10 genera and environmental factors.

Further alignment and annotation based on the short‐read showed that Dinophyceae dominated during the entire year, accounting for 55.5%–90.0% of the total phytoplankton (Figure 2a), and Gymnodiniales contributed the most (Figure 2b). In addition, the contents of Prymnesiophyceae, Bacillariophyceae, and Prasinophyceae were also high, accounting for 5.4%–27.9%, 0.1%–20.8%, and 0%–9.6% of the phytoplankton abundance, respectively (Figure 2a). Among the top 20 genera, 12 belonged to Dinophyceae, three belonged to Prymnesiophyceae, two belonged to Bacillariophyceae, two belonged to Prasinophyceae and one belonged to Cryptophyceae (Figure 2c). Among Dinophyceae, *Karlodinium* had the highest average relative abundance throughout the year, accounting for 19.8% of the total abundance of phytoplankton; *Amoebophrya* followed, accounting for 11.8% of the total abundance of phytoplankton; and *Noctiluca* mainly appeared in February and March 2017, and its relative abundance was the highest in March 2017, accounting for 25.9% of phytoplankton. Prymnesiophyceae mainly consisted of *Chrysochromulina* in August 2017, September 2016, and November 2016, while *Phaeocystis* predominated from December 2016 to March of the following year. In June and November 2016, Bacillariophyceae mainly consisted of *Leptocylindrus*, but in January and February 2017, it mainly consisted of *Chaetoceros*. Prasinophyceae was dominated by *Micromonas* in November 2016 and by *Ostreococcus* in March and June 2017. Redundancy analysis revealed that phosphate had the greatest impact on the variations of top 10 genera, followed by ammonium and silicate (Figure 3). Notably, *Ostreococcus* was most closely related to DIN, and *Phaeocystis* was closely related to DIN/DIP (Figure 3).

The full‐length sequences of SSU rRNA for each sample were obtained by the PacBio sequencing approach. Through comparative analysis, a total of 1087 OTUs were obtained, of which 512 OTUs were annotated to phytoplankton, and 147 OTUs were identified to the category level (PID > 97%), including 118 species that contained 41 species of Dinophyceae, 37 species of Bacillariophyceae, 14 species of Prasinophyceae, 10 species of Prymnesiophyceae, 8 species of Cryptophyceae, 3 species of Chrysophyceae, 2 species of Raphidophyceae, 2 species of Dictyochophyceae, and 1 species of Pinguiophyceae (Table S1). Most of the compared sequences were longer than 1800 bp, and the types with PID greater than 97% were selected. Notably, a considerable number of OTUs that were not annotated to the species level were annotated to parasitic dinoflagellates and syndinoflagellates.

Further analysis showed that the highly abundant genera, including *Karlodinium*, *Amoebophrya*, *Phaeocystis*, *Noctiluca*, *Lepidodinium*, *Karenia*, *Prorocentrum*, *Chrysochromulina*, *Ostreococcus*, and *Chaetoceros*, identified through short‐read metabarcoding yielded relevant species in the long‐read metabarcoding, including *Karlodinium micrum*, *Lepidodinium viride*, *Noctiluca scintillans*, *Prorocentrum micans* and *Ostreococcus* sp. *“lucimarinus*.” Many species of Gymnodiniales in dinoflagellates were also identified, including *K*. *micrum*, *L*. *viride*, *Gymnodinium aureolum*, *Gymnodinium beii*, *Gymnodinium* cf. *nolleri*, *Gymnodinium heterostriatum*, *Gymnodinium* sp., *Gyrodinium* cf. *gutrula*, and *Gyrodinium jinhaense*. Based on the long‐read metabarcoding, *Amoebophrya* sp. *ex Alexandrium affine*, *Amoebophrya* sp. *ex Margalefidinium polykrikoides*, *Amoebophrya* sp. *ex Ostreopsis lenticularis*, *Amoebophrya* sp. “*Dinophysis*,” and other species were identified, and these were also verified by the full‐length sequence results to be parasitic dinoflagellates, with high contents obtained from short‐read metabarcoding.

### 3.2 Phylogenetic analysis of the dominant genera of phytoplankton

**FIGURE 4:**
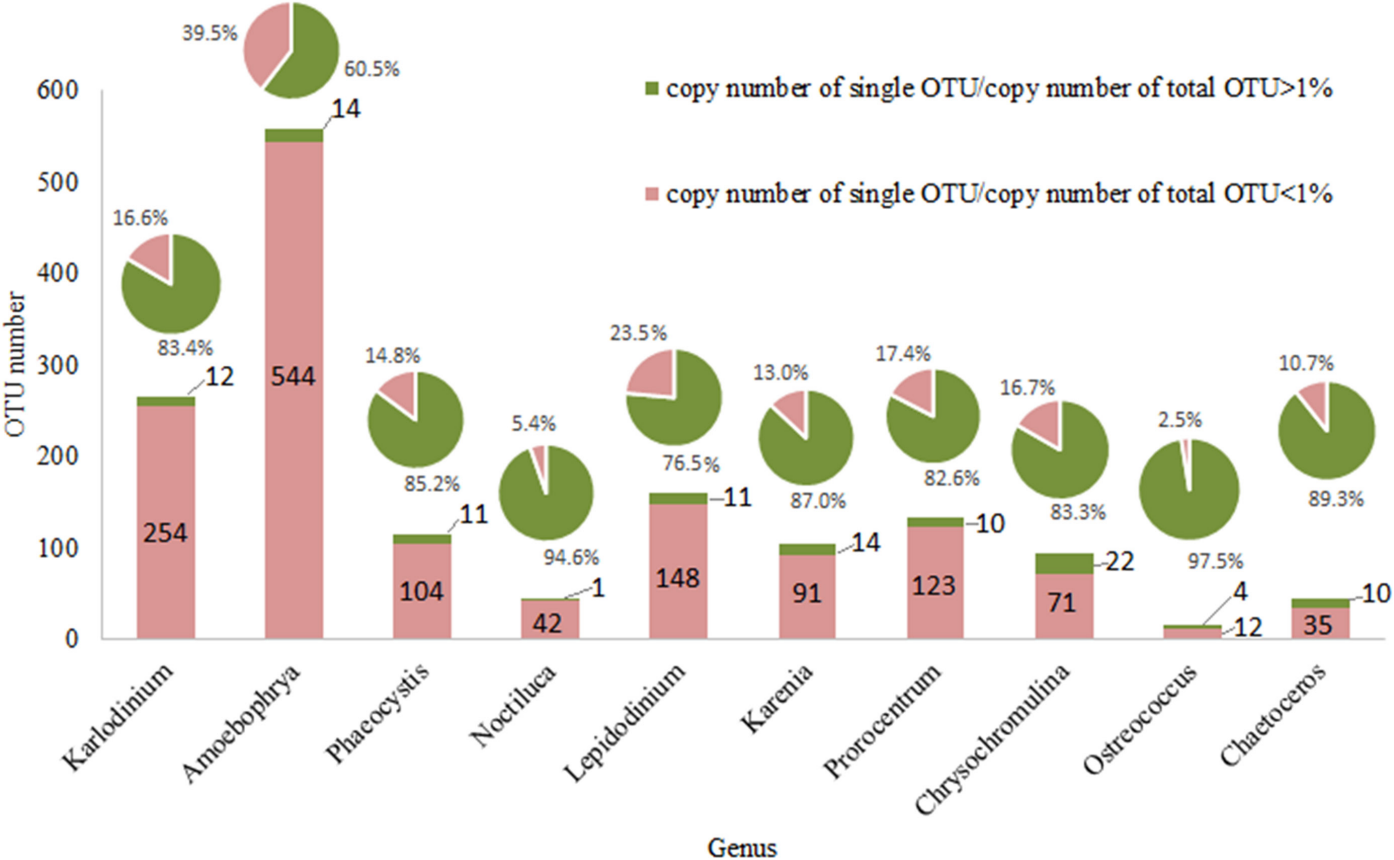
OTU number and relative abundance of the top 10 genera. Among them, the histogram in red represents the total number of OTUs whose copy number for a single OTU exceeded 1% of its total copy number, and green represents the total number of OTUs whose copy number for a single OTU was less than 1% of its total copy number. The pie chart represents the proportion of the corresponding OTU copy number to the total abundance of the genus.

As the top 10 genera already accounted for 66.4% of the total phytoplankton abundance, we further analyzed the numbers of OTU annotated to each of these genera. The results showed that each genus contained a variety of OTUs, with *Amoebophrya* the most (558) and *Ostreococcus* the least (16; Figure 4). Among these genera, the OTU types whose copy number for a single OTU exceeded 1% of its total copy number were the most common for *Chrysochromulina* (22) and the least common for *Noctiluca* (1; Figure 4). Except for *Noctiluca*, the other dominant genera contained multiple high abundance OTUs, showing a high degree of heterogenecity. Moreover, the abundance of the dominant OTUs above accounted for 60.5%–97.5% of the total copies (Figure 4). To further investigate the intragenus diversity of the dominant genera above, the V4 region sequences of high abundance OTUs were extracted. The sequences of known representative species of the above genera were selected from the SILVA database for phylogenetic analysis, and the above nine genera were divided into two categories: Dinophyceae, including *Karlodinium*, *Amoebophrya*, *Lepidodinium*, *Karenia*, and *Prorocentrum*, and other algae, including *Phaeocystis*, *Chrysochromulina*, *Ostreococcus*, and *Chaetoceros*.

**FIGURE 5:**
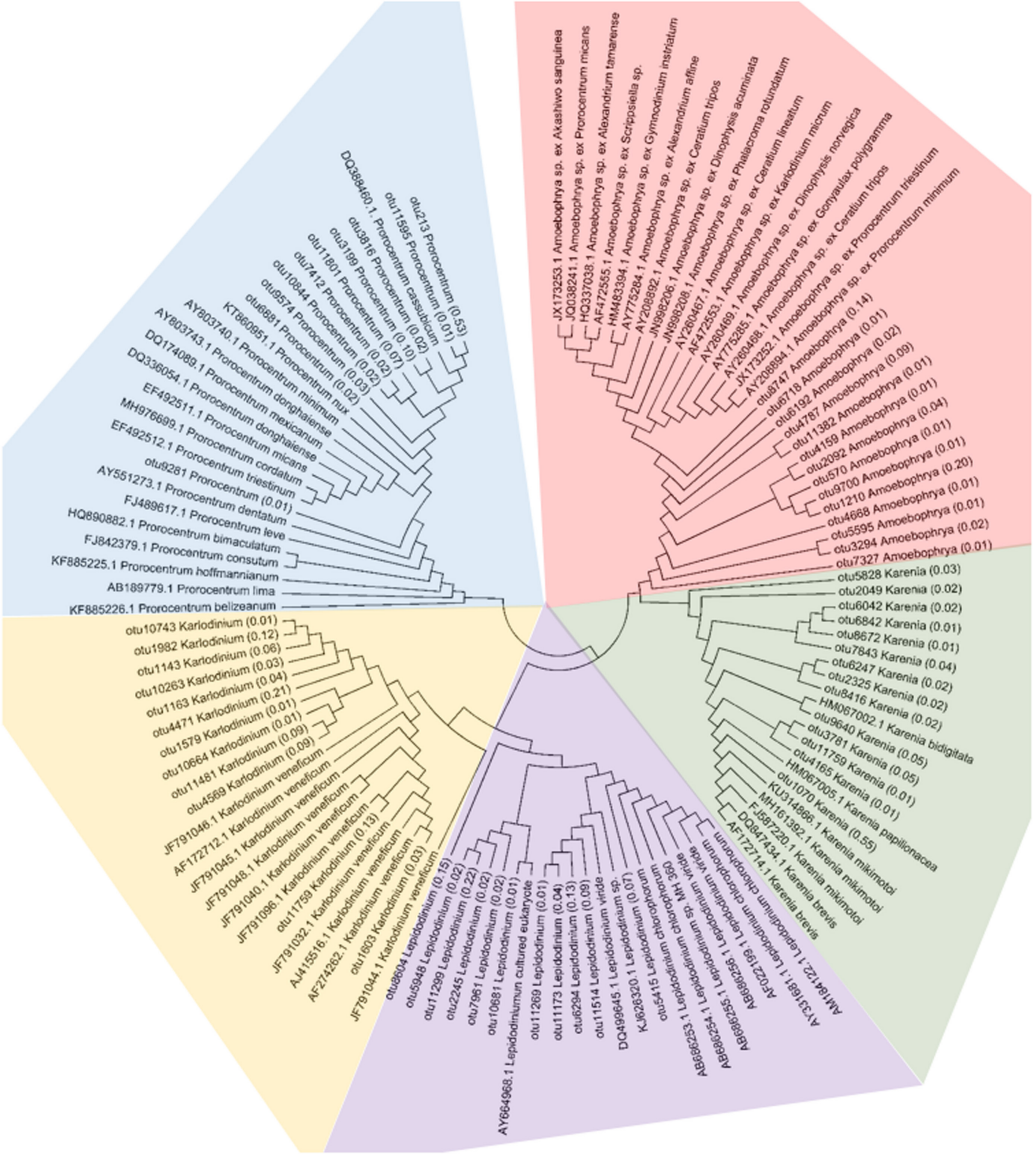
Molecular phylogenetic analysis of *Karlodinium*, *Amoebophrya*, *Lepidodinium*, *Karenia*, and *Prorocentrum*. The tree with the highest log likelihood (−6552.9313) is shown. The analysis involved 120 nucleotide sequences. All positions containing gaps and missing data were eliminated. There were a total of 241 positions in the final dataset. The numbers in brackets represent the relative content of the OTU in the genus.

**FIGURE 6:**
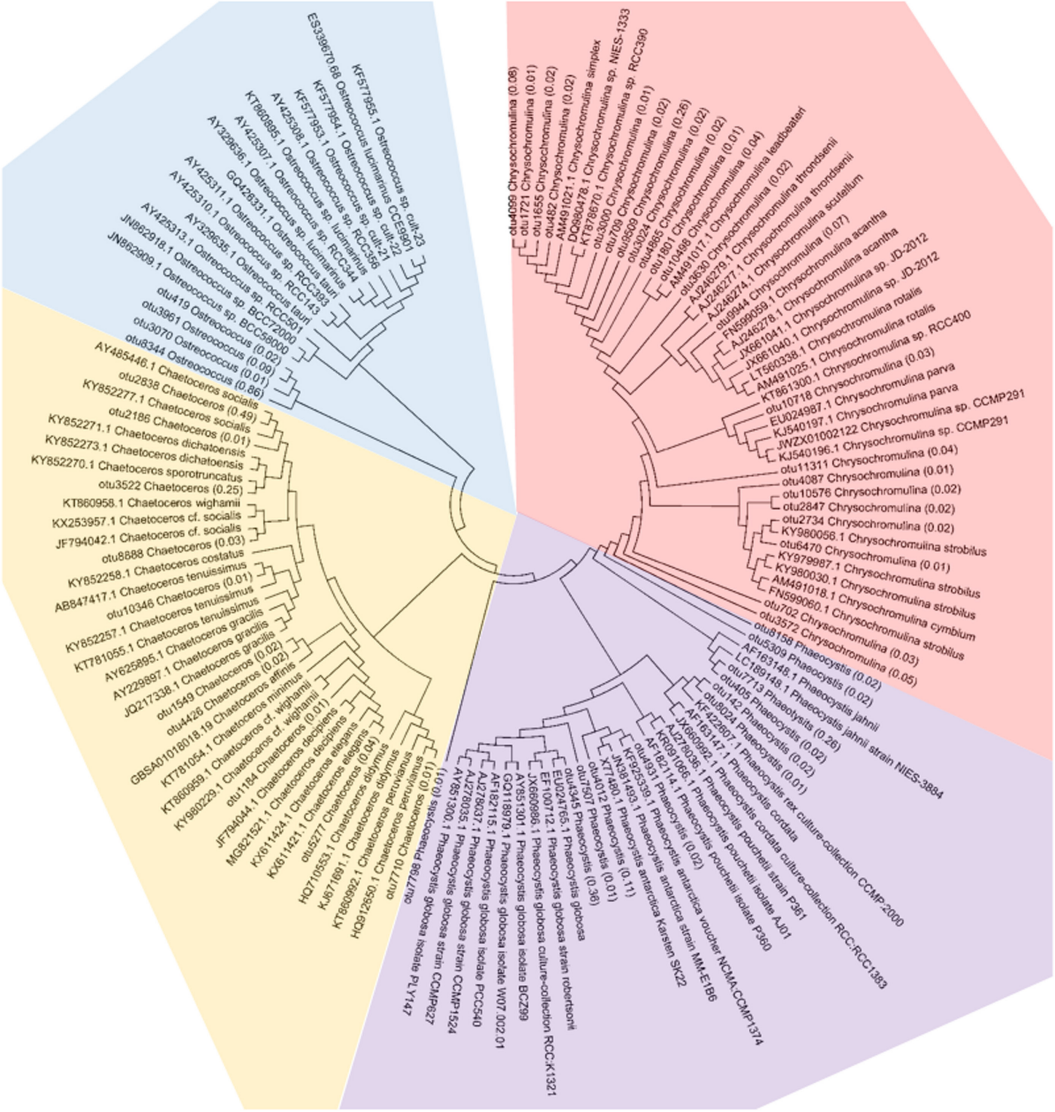
Molecular phylogenetic analysis of *Phaeocystis*, *Chrysochromulina*, *Ostreococcus* and *Chaetoceros*. The tree with the highest log likelihood (−4137.9276) is shown. The analysis involved 132 nucleotide sequences. All positions containing gaps and missing data were eliminated. There were a total of 304 positions in the final dataset. The numbers in brackets represent the relative content of the OTU in the genus.

Based on the short‐read metabarcoding, we established phylogenetic relationships among 120 dinoflagellate sequences, including the *Karlodinium*, *Amoebophrya*, *Lepidodinium*, *Karenia*, and *Prorocentrum* OTUs obtained in this study and the representative sequences from the SILVA database. Each of the above genera formed an independent cluster. For *Karlodinium*, OTU1603 and OTU11759 were closest to the known sequences AF274262.1 and JF791040.1, respectively, altogether accounting for 15.7% of the total amount of *Karlodinium*. The remaining 10 OTUs, including OTU4471, which had the highest abundance, were not directly clustered with the known sequences, indicating the high diversity of *Karlodinium*. Similar to *Karlodinium*, although all of the *Amoebophrya* sequences clustered well, the OTUs of 14 *Amoebophrya* obtained in this study were not closely related to the known sequences. The 11 OTUs of *Lepidodinium* were scattered in several branches. *Karenia*'s 14 OTUs also showed diversity. The 10 OTUs of *Prorocentrum* were neither clustered with known common species such as *Prorocentrum donghaiense* nor directly clustered with *Prorocentrum minimum* and *P*. *micans* (Figure 5). The phylogenetic relationships among the 11 OTUs of *Phaeocystis* found in this study and several known *Phaeocystis* species, such as *Phaeocystis cordata*, *Phaeocystis jahnii*, *Phaeocystis pouchetii*, *P*. *globosa*, *Phaeocystis antarctica*, and *Phaeocystis rex*, were constructed. The 22 OTUs of *Chrysochromulina* showed high diversity. The OTUs of the four *Ostreococcus* species were scattered in known groups. The 10 OTUs related to *Chaetoceros* were also scattered in several groups. For example, OTU2838 (48.7%), which had the highest abundance, was clustered in *Chaetoceros socialis*, and OTU3522 (24.6%) was clustered in *Chaetoceros sporotruncatus* (Figure 6).

Further analysis shows that the high abundance HAB genera, including *Karlodinium*, *Phaeocystis*, *Noctiluca*, *Lepidodinium*, *Karenia*, *Prorocentrum*, *Chrysochromulina*, and *Chaetoceros*, identified through short‐read metabarcoding had related species in the long‐read metabarcoding (Du Yoo et al., 2017; Guo, 2004; Jiao et al., 2010; Tomaru et al., 2009), which included *K*. *micrum*, *L*. *viride*, *N*. *scintillans*, *P*. *micans*, etc (Table S1).

In this study, although the relative abundance of Bacillariophyceae was not high overall, its diversity was high. A total of 37 species of Bacillariophyceae were found throughout the year, of which *Chaetoceros* was the highest in both abundance and intragenus diversity. A total of seven species of *Chaetoceros* were identified by the full‐length sequencing approach, including *C*. *socialis*, *Chaetoceros eibenii*, *Chaetoceros contortus*, *Chaetoceros pseudocurvisetus*, *Chaetoceros elegans*, *Chaetoceros* cf. *lauderi*, and *Chaetoceros* sp., which was consistent with the results of short‐read metabarcoding. In this study, *P*. *cordata* and *P*. *jahnii* were identified. In addition, a variety of *Chrysochromulina* were found, including *Chrysochromulina campanulifera*, *Chrysochromulina cymbium*, *Chrysochromulina leadbeateri*, and *Chrysochromulina scutellum*, which was consistent with the short sequencing results.

### 3.3 Comparisons between long‐read and short‐read metabarcoding

In this study, the results of the two metabarcoding approaches were also compared. The number of clean reads generated by MiSeq was 38,911, and that of PacBio was 58,666. The former had a large amount of sequencing data with approximately 6.6 times more than that of the latter and covered more phytoplankton information. However, the average read length of MiSeq was approximately 398 bp, while that of PacBio was approximately 1789 bp, so the average read length of PacBio was approximately 4.5 times that of MiSeq (Table 3). After removing rare OTUs, a total of 3595 OTUs were obtained by MiSeq sequencing. Although 313 OTUs were annotated to the species level, the reliability was not high due to the relatively short fragments length. In contrast, 1087 OTUs were obtained from PacBio sequencing, and the 148 OTUs annotated to the species level were more accurate, especially for identifying specific species.

**TABLE 3:** Parameter comparisons between short‐read and long‐read metabarcoding.

Parameter	Short‐read	Long‐read
Clean reads	384,911	58,666
Average read length (bp)	398	1789
After filtering rare OTU	3595	1087
OTU annotated to phytoplankton	512	512
OTU annotated to species level		148

**FIGURE 7:**
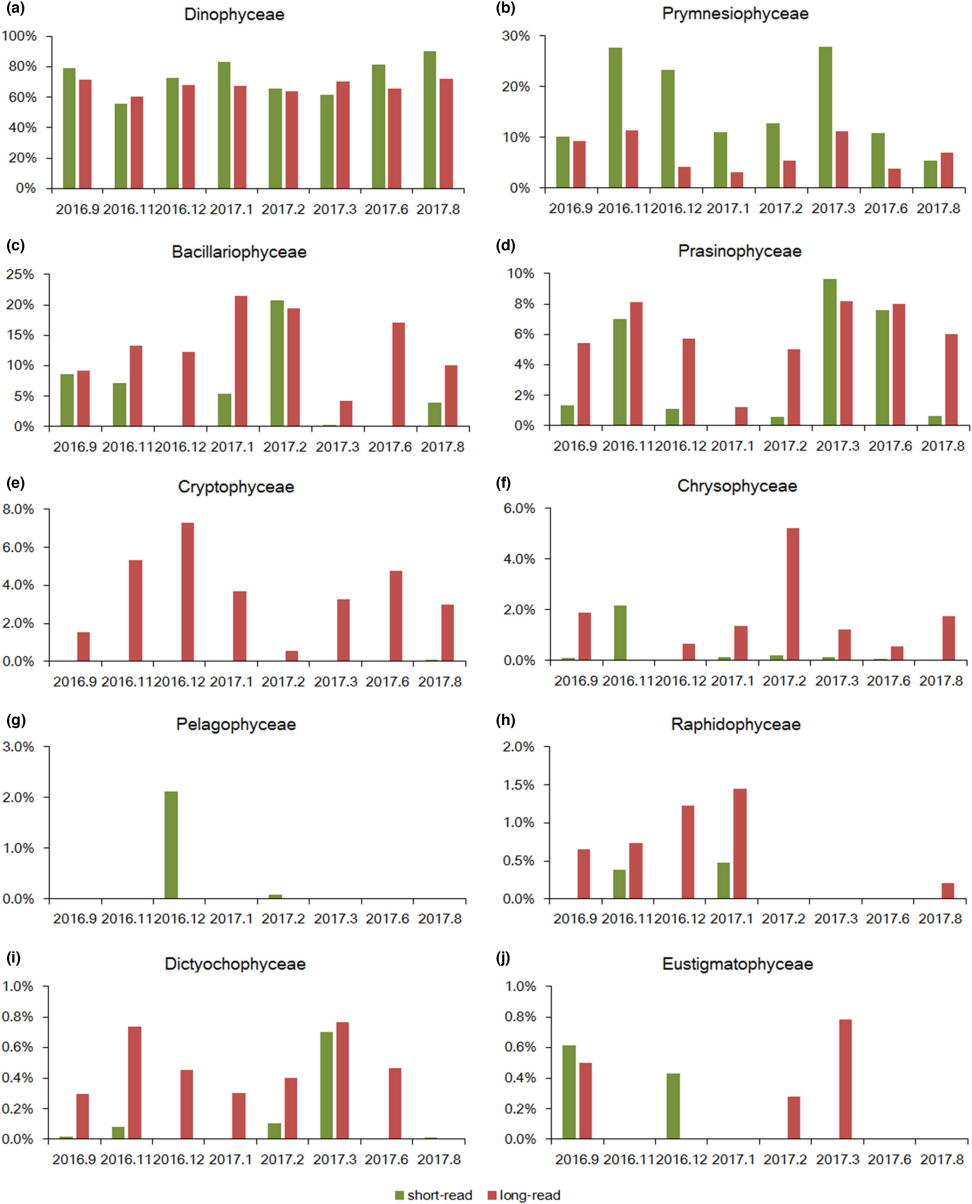
Comparison between short‐read and long‐read metabarcoding at the class level. (a) Dinophyceae (b) Prymnesiophyceae (c) Bacillariophyceae (d) Prasinophyceae (e) Cryptophyceae (f) Chrysophyceae (g) Pelagophyceae (h) Raphidophyceae (i) Dictyochophyceae, and (j) Eustigmatophyceae.

In general, the phytoplankton species identified in this study based on the short‐read and long‐read metabarcoding were similar at the class level; that is, they all included Dinophyceae, Bacillariophyceae, Prasinophyceae, Prymnesiophyceae, Chrysophyceae, Raphidophyceae, and Cryptophyceae. However, compared with the results of short‐read metabarcoding, the proportions of Dinophyceae and Prymnesiophyceae identified by long‐read metabarcoding decreased from mean values of 73.6% and 16.1% to 67.4% and 6.9%, respectively (Figure 7a,b). The proportions of identified Bacillariophyceae and Cryptophyceae increased from 5.8% and 0.017% to 13.4% and 3.7%, respectively (Figure 7c,e). In general, for Cryptophyceae, Chrysophyceae, Raphidophyceae, Dictyochophyceae and Eustigmatophyceae with low relative abundance, the detection rate of long‐read metabarcoding is higher, especially for Cryptophyceae and Chrysophyceae.

The identified phytoplankton community based on the two metabarcoding approaches were similar at the class level, but quite different at the genus level. Firstly, the identified genera compositions and their relative abundances varied. Among the top 20 genera identified by the two metabarcoding approaches, only 6 genera were the same, namely, *Karlodinium*, *Amoebophrya*, *Phaeocystis*, *Chaetoceros*, *Micromonas*, and *Leptocylindrus* (Table 4). Secondly, the degrees of phytoplankton diversity varied. Short‐read metabarcoding showed more diversity below genus level. In addition, short‐read metabarcoding showed that most genera among top 10 contained multiple OTUs, indicating their high diversity below genus level (Figure 3). However, only 3 genera based on long‐read metabarcoding identified multiple species, namely, *Amoebophrya* (identified six species), *Chrysochromulina* (identified six species), and *Chaetoceros* (identified seven species; Table S1).

**TABLE 4:** Comparison of top 20 genera between short‐read metabarcoding and long‐read metabarcoding at the genus level.

Rank	Genus (relative abundance) by short‐read metabarcoding	Genus (relative abundance) by long‐read metabarcoding
1	***Karlodinium*** (21.81%)	*Syndiniales*_Group_II (16.07%)
2	***Amoebophrya*** (19.83%)	***Amoebophrya*** (13.43%)
3	***Phaeocystis*** (11.82%)	*Syndiniales*_Group_I (5.32%)
4	*Noctiluca* (7.32%)	*Azadinium* (4.48%)
5	*Lepidodinium* (5.58%)	***Chaetoceros*** (3.12%)
6	*Karenia* (5.47%)	***Micromonas*** (2.42%)
7	*Prorocentrum* (5.12%)	*Syndiniales*_Group_III (2.13%)
8	*Ostreococcus* (4.02%)	*Gyrodinium* (1.84%)
9	*Chrysochromulina* (3.68%)	*Thalassiosira* (1.74%)
10	*Teleaulax* (1.81%)	*Skeletonema* (1.74%)
11	***Chaetoceros*** (1.76%)	*Protoperidinium* (1.74%)
12	*Takayama* (1.66%)	***Karlodinium*** (1.56%)
13	*Syracosphaera* (1.15%)	*Erythropsidinium* (1.50%)
14	*Brachidinium* (1.14%)	*Fragilariopsis* (1.48%)
15	*Gymnodinium* (1.05%)	*Neoceratium* (1.04%)
16	*Scrippsiella* (0.99%)	***Leptocylindrus*** (0.98%)
17	***Micromonas*** (0.90%)	*Haplozoon* (0.91%)
18	*Peridiniopsis* (0.83%)	*Gonyaulax* (0.88%)
19	***Leptocylindrus*** (0.81%)	*Geminigera* (0.85%)
20	*Warnowia* (0.68%)	***Phaeocystis*** (0.85%)

*Note*: Genera in bold font means both identified in the two metabarcoding approaches.

## 4 DISCUSSION

### 4.1 Phytoplankton species in the coastal waters of Beibu Gulf

Previous studies on phytoplankton species and communities in the Beibu Gulf were mostly based on microscopic observation approaches. In 2011, diatoms were the main group in the water sample after filtration with a 77 μm net in the northern area of the Beibu Gulf, and the dominant species were *Thalassiosira subtilis*, *L*. *danicus*, *Bacillaria paradoxa*, and *Pseudonitzschia pungens* in the spring and *Thalassiosira* sp. in the summer (Wang et al., 2015). In addition to microscopy, an approach based on pigment detection showed that the main phytoplankton groups from September 2016 to August 2017 were diatoms, haptophytes, cryptophytes, prasinophytes, dinoflagellates, chlorophytes, etc. (Wang, Kong, et al., 2021). A molecular biological analysis approach based on the rbcL fragment (550 bp) showed that Bacillariophyceae, Coscinodiscophyceae, Fragilariophyceae, and Mediophyceae were the most abundant groups in the periods before (November 2017), during (December 2017) and after (February 2018) the HAB event involving *P*. *globosa* (Xu et al., 2022). The time period of this study coincides with that of the investigation by Wang, Kong, et al. (2021), which used the pigment approach. The phytoplankton categories obtained were similar, but this study provides more detailed results.

In the above study, as the water samples were filtered by a 77 μm net, no information on small phytoplankton species was obtained. However, this size fraction is also an important component of the phytoplankton community. Phytoplankton of 0.68–200 μm were obtained in this study, which were mainly dinoflagellates, accounting for 55.5%–90.0% of the annual phytoplankton. These included *Karlodinium*, which is difficult to preserve with ordinary fixatives, and *Amoebophrya*, which has a small cell size (Chen et al., 2019). These two genera are difficult to accurately identify and count by microscopic observation approaches even though they account for up to 51.3% of the total abundance of phytoplankton. *Noctiluca* accounted for a relatively high proportion in February and March 2017, accounting for 17.9% and 25.9% of the total abundance of phytoplankton, respectively. In this study, a large number of parasitic dinoflagellate *Amoebophrya* were identified in the Beibu Gulf, which were detected at eight time points during this study, and their relative content accounted for 6.23%–23.45% of the total phytoplankton. Based on the long‐read metabarcoding alignment results, *Amoebophrya* sp. *ex Alexandrium affine*, *Amoebophrya* sp. *ex M*. *polykrikoides*, *Amoebophrya* sp. *ex Ostreopsis lenticularis*, *Amoebophrya* sp. “*Dinophysis”* and other species were identified. Because *Amoebophrya* can infect specific dinoflagellates, it can regulate the occurrence of HABs (Mazzillo et al., 2011). Recent studies have found that *Amoebophrya* widely exists in the coastal areas of China (Chen et al., 2019). Studies based on the SSU rRNA V4 region show that *Amoebophrya* is dominant in the phytoplankton community of Jiaozhou Bay, while it was overlooked by previous microscopic approaches (Chen et al., 2022). By combining morphological observations with sequencing analyses based on the SSU rRNA V2–V3 region, it was found that parasitic dinoflagellates were dominant in the Yellow Sea and East China Sea, which was overlooked in morphological studies (Liu et al., 2017). The *Amoebophrya* identified in this study did not completely match the known species but formed a cluster of its own, showing that it is unique.

Although the relative abundance of diatoms in this study was not high, their diversity was high. As many as 37 diatom species were found throughout the year, among which *Chaetoceros* had the highest diversity in both abundance and genus, and it showed the highest diversity in February 2017. The short‐read metabarcoding results showed that there were 10 OTUs of *Chaetoceros* whose copy number for a single OTU exceeded 1% of its total copy number, and 7 species of *Chaetoceros* were identified by long‐read metabarcoding, including *C*. *socialis*, *C*. *eibenii*, *C*. *contortus*, *C*. *pseudocurvisetus*, *C*. *elegans*, *Chaetoceros* cf. *lauderi*, and *Chaetoceros* sp.

In addition to the above dinoflagellates and diatoms, this study also identified many kinds of small algae that are difficult to identify by traditional microscopic approaches, including those belonging to picophytoplankton Prasinophyceae: *Ostreococcus* (Demir‐Hilton et al., 2011), *Micromonas* (Worden et al., 2009), and *Bathycoccus* (Moreau et al., 2012). *Ostreococcus* is the smallest eukaryote that can live freely (Courties et al., 1994). In this study, its content was the highest in March 2017, accounting for 8.98% of the total phytoplankton, followed by June 2017, accounting for 5.46% of the total phytoplankton. *Ostreococcus* sp. “*lucimarinus*” was identified based on the results of long‐read metabarcoding. *Micromonas* had the highest content in November 2016, accounting for 6.00% of the total phytoplankton, followed by June 2017, accounting for 1.40% of the total phytoplankton. As a green algae, *Micromonas* has been proven to have the ability to take up small particles (McKie‐Krisberg & Sanders, 2014). *Micromonas bravo* and *Micromonas* sp. were identified based on long‐read metabarcoding. The other algae, such as *G*. *beii*, were smaller than 10 μm (Spero, 1987). Another microalgae that is difficult to identify and completely preserve by microscopic approaches is *Phaeocystis*. The size of *Phaeocystis* in a single cell is 3–8 μm. However, when *Phaeocystis* forms colonies, it is difficult to preserve with common fixatives.

Another feature found in this study was the frequent occurrence of intragenus diversity. Previous studies based on the LSU rDNA D1–D2 region showed that there was distinct genetic diversity in *P*. *globosa* in the Beibu Gulf, and there were clear genetic differences among the strains of *P*. *globosa* isolated from different years (Hu et al., 2019). Furthermore, based on morphological and molecular taxonomic studies, *Akashiwo sanguinea* is highly diverse near Weizhou Island in the Beibu Gulf (Xu et al., 2020), which is similar to the conclusion of this study. In addition to the genus‐level analyses, based on long‐read metabarcoding, this study identified a variety of new species in the Beibu Gulf. The dinoflagellate *Paragymnodinium shiwhaens*e was first recorded in 2010 (Kang et al., 2010).

### 4.2 HAB species and their ecological characteristics in the Beibu Gulf

Previous studies have reported many kinds of HAB organisms in the Beibu Gulf. Prior to 2005, cyanobacteria, such as *T*. *erythraeum*, *T*. *hildebrandtii*, *M*. *aeruginosa*, and *M*. *aquatica*, were dominant. While since 2005, HAB types in the Beibu Gulf have become more diverse and include dinoflagellates, diatoms, and other HAB species, including *Noctiluca* sp., *P*. *globosa* and *L*. *danicus*. Particularly after 2014, *P*. *globosa* caused HABs in many places in the Beibu Gulf (Jiang, 2019; Luo et al., 2016; Wang, Song, et al., 2021). Based on short‐read metabarcoding, a large number of HAB genera were found in this study. Among the top 20 genera, 15 genera, including *Karlodinium*, *Phaeocystis*, *Noctiluca*, *Lepidodinium*, *Karenia*, *Prorocentrum*, *Chrysochromulina*, *Chaetoceros*, *Teleaulax*, *Takayama*, *Syracosphaera*, *Gymnodinium*, *Scrippsiella*, *Peridiniopsis*, and *Leptocylindrus*, are HAB genera (Du Yoo et al., 2017; Guo, 2004; Jiao et al., 2010; Tomaru et al., 2009), and these genera account for 47.3%–71.5% of the relative content of phytoplankton over the entire year, which reflects both the high diversity and abundance of HAB in the coastal waters of the Beibu Gulf.

Most of the above high abundance HAB genera have a special life history. For example, a variety of dinoflagellates, including *Karlodinium*, *Karenia*, *Gymnodinium*, and *Scrippsiella*, can form resting cysts, which are key factors in their algal blooms and geographical spread (Liu et al., 2020a, 2020b; Tang et al., 2021). In addition to single‐celled species, *P*. *globosa*, which has bloomed frequently in the Beibu Gulf in recent years, also has a special life stage of forming colonies. *P*. *globosa* was present year‐round in this study, and its content was high in December 2016, January 2017, February 2017, and March 2017, accounting for 6.78%–20.66% of the total phytoplankton, which is similar to the results based on the number of colonies during the same period (He et al., 2019). Since the cell size of phytoplankton collected in this study was 0.68–200 μm, large *Phaeocystis* colonies were filtered out, and a certain amount of biomass was lost when the large colonies showed high densities. In addition to *P*. *globosa*, *P*. *cordata* and *P*. *jahnii* were also identified based on long‐read metabarcoding, and this was the first record of these two *Phaeocystis* species in the Beibu Gulf.

In addition to the above species that have special life histories, the HAB genera in the Beibu Gulf also have various modes of nutrient utilization. Except for *Amoebophrya*, the top 10 genera with high relative abundance in this study included *Karlodinium*, *Phaeocystis*, *Noctiluca*, *Lepidodinium*, *Karenia*, *Prorocentrum*, *Chrysochromulina*, *Ostreococcus*, and *Chaetoceros*, and the nutritional mode of these algae is mixotrophy (Burkholder et al., 2008; Hernandez‐Ruiz et al., 2018; Morando & Capone, 2018; Stoecker, 1999). Notably, in recent years, an increasing number of green algae and diatoms that are commonly considered phototrophic have been found to belong to mixotrophy (McKie‐Krisberg & Sanders, 2014). Mixotrophy provides an important way for algal cells to adapt to environmental nutritional conditions, which is important for the formation and development of algal blooms (Burkholder et al., 2008; Stickney et al., 2000; Stoecker et al., 2017). The results of redundancy analysis showed that phosphate had the greatest impact on species composition, followed by ammonium and silicate. Notably, *Ostreococcus* was most closely related to DIN, and *Phaeocystis* was closely related to DIN/DIP (Figure 3).

Importantly, a considerable fraction of the above algae is toxic and potentially harmful to aquaculture organisms. For example, *Karlodinium* can produce karlotoxins, which are toxic to fish, and karlotoxins also play a role in the process of feeding *Oxyrrhis marina* (Adolf et al., 2007; Place et al., 2012). *Karenia* can produce brevetoxin, which is also toxic to fish (Landsberg et al., 2009; van Deventer et al., 2012). *Phaeocystis* can produce a hemolytic toxin, which also has toxic effects on fish during outbreaks and extinctions of HAB events (Peng et al., 2005; Smith et al., 2014). *Chrysochromulina* can also produce toxins, which can lead to large‐scale fish mortality (Stabell et al., 1993). These toxic algae could not be overlooked, as their proportions in the total phytoplankton were relatively high, varying from 19.1% to 51.1% within the top 20 genera. Even for nontoxic algae such as *Noctiluca*, blooms can cause hypoxia, leading to the death of fish and benthic animals (Cardoso, 2012).

Previous studies have also demonstrated HAB diversity in other areas based on metabarcoding approaches. For example, 42 HAB species, especially fragile and small species, were identified in the South China Sea (Wang et al., 2022). Eighty‐six HAB species, including 45 Dinoflagellata and 37 Ochrophyta, were identified in the Changjiang Estuary (Cui et al., 2021). In the East China Sea, 58 HAB species were identified, with *P*. *donghaiense*, *Lebouridinium glaucum* and *N*. *scintillans* showing high abundance (Chen et al., 2021). In the Bohai Sea, 21 potential HAB species were dinoflagellates (Xu et al., 2017), and high relative abundances of the ichthyotoxic phytoplankton species *Vicicitus globosus* (Dictyochophyceae) were also detected (Huang et al., 2021). In Jiaozhou Bay, 28 HAB species were detected, mainly including 17 species of Bacillariophyta and 8 species of Dinoflagellata (Liu, Gibson, et al., 2020). This study detected 37 HAB species in the Beibu Gulf, of which 15 species belonged to Dinophyceae and 13 species belonged to Bacillariophyceae, and the fragile *Gymnodinium* dominated (Table S1).

### 4.3 Comparisons between short‐read and long‐read metabarcoding for marine phytoplankton

This study also compared the phytoplankton community based on short‐read and long‐read metabarcoding approaches. The results obtained were slightly different depending on taxonomy level. On the one hand, this may be due to the bias of primers (Bai et al., 2019; Giebner et al., 2020; Nichols et al., 2018); on the other hand, it may be affected by the alignment process and influenced by the sequencing depth. Previous studies have indicated that different primers have different biases for amplified fragments during algal species identification. For example, the V2 region has the highest taxonomic resolution for dinoflagellates (Ki, 2012), while the V4 region is more suitable for the identification of diatoms (Luddington et al., 2012).

Unlike phytoplankton community at the class level, the results of the two metabarcoding approaches were quite different below the genus level. At present, short‐read metabarcoding approaches using the V4 or V9 region is still common for phytoplankton identification (dos Santos et al., 2017; Ribeiro et al., 2018). However, due to the restricted sequence length, short‐read metabarcoding approaches cannot accurately identify phytoplankton in some closely related species. This situation can be greatly improved by replacing short‐read sequence with long‐read sequence, as the sensitivity and accuracy of species identification increases with increasing sequence read length (Yarza et al., 2014). By combining these two metabarcoding approaches, phytoplankton community in the Beibu Gulf can be more systematically studied.

## 5 CONCLUSIONS

Metabarcoding approaches based on short‐read and long‐read were employed in this study to investigate the phytoplankton community in the coastal waters of the Beibu Gulf from September 2016 to August 2017. The biodiversity of phytoplankton in this area is high, dominated by Dinophyceae, especially Gymnodiniales, which compensates for Gymnodiniales being difficult to preserve by using fixative in the traditional microscopic approach. A large number of small phytoplankton (Prymnesiophyceae, Prasinophyceae, etc.) with high abundance were identified, which also supplemented the limitation of the microscopic approach in the lack of discrimination of small phytoplankton. A large number of algae belonging to the same genera and different species were identified, which made up for the interspecific differences that were difficult to distinguish under the light microscope. Ninety‐eight species of algae were reported for the first time in the Beibu Gulf, which increased the list of phytoplankton recorded in the basic resources of this area.

Harmful algal bloom species with high diversity and abundance were revealed in the coastal waters of the Beibu Gulf. Most of the relatively abundant genera belonged to HAB species. Among the top 20 genera, 15 genera could cause HABs, and these genera accounted for 47.3%–71.5% of the relative abundance of phytoplankton over the entire year. The phylogenetic analysis results show that the above dominant genera had a high degree of intragenus diversity, reflecting both the high diversity and abundance of HAB species in the study area. The special life history of forming cysts or colonies and the advantages of multiple nutrition strategies may be the key factors affecting these HAB species. Due to their toxicity and special life history stages, the potential impacts of these species on aquaculture and nuclear power plant safety in the Beibu Gulf are noteworthy.

This study also compared short‐read and long‐read metabarcoding approaches to study marine phytoplankton communities. They were essentially similar at the class level, that is, they both identified Dinophyceae, Bacillariophyceae, Prasinophyceae, Prymnesiophyceae, Chrysophyceae, Raphidophyceae, and Cryptophyceae. However, compared with the short‐read metabarcoding, the proportions of Dinophyceae and Prymnesiophyceae identified by long‐read metabarcoding were higher, while the proportions of Bacillariophyceae and Cryptophyceae identified were lower. Notably, the outcomes of the two metabarcoding approaches were quite different below the genus level. These distinctions may have been caused by differences in primer bias, database comparisons and sequencing depth.

In summary, short‐read and long‐read metabarcoding were combined for the first time, yielding both high richness from short‐reads and highly accurate species identification from long‐reads. The phytoplankton community and the characteristics of HAB species in the Beibu Gulf were systematically described, providing a basis for clarifying the potential impact of HAB species in the Beibu Gulf.

## AUTHOR CONTRIBUTIONS

**Liyan He:** Data curation (equal); formal analysis (equal); funding acquisition (equal); resources (equal); writing – original draft (equal); writing – review and editing (equal). **Zhiming Yu:** Conceptualization (equal); funding acquisition (equal); supervision (lead); writing – review and editing (equal). **Xin Xu:** Formal analysis (supporting); resources (equal). **Jianan Zhu:** Formal analysis (supporting); resources (equal). **Yongquan Yuan:** Resources (equal). **Xihua Cao:** Resources (equal). **Xiuxian Song:** Conceptualization (equal).

## CONFLICT OF INTEREST STATEMENT

The authors declare no conflicts of interest.

## Supporting information


**Table S1.** Phytoplankton species identified by long‐read metabarcoding.
**Table S2.** Phytoplankton genera and OTU number identified by short‐read metabarcoding.

## Data Availability

The raw sequences are available on NCBI, with short‐read sequences (BioProject ID: PRJNA854303) and long‐read sequences (BioProject ID: PRJNA854304).
