# Supplementary material for: Metabarcoding analysis identifies high diversity of harmful algal bloom species in the coastal waters of the Beibu Gulf
Source: Ecol Evol. 2023 May 22;13(5):e10127. doi: 10.1002/ece3.10127 (PMC10202623; doi:10.1002/ece3.10127)
Supplement: Supplementary file 1 — Table S1. Phytoplankton species identified by long‐read metabarcoding. Table S2. Phytoplankton genera and OTU number identified by short‐read metabarcoding. [file ECE3-13-e10127-s001.docx]

**Supplementary table 1** Phytoplankton species identified by long-read metabarcoding

| **OTU ID** | **Query length**  **(bp)** | **Division** | **Class** | **Species** | **NCBI accession** | **PID (%)** | **Acc. Len**  **(bp)** | **Reported** | **HABs** | **HABs evidence** |
| --- | --- | --- | --- | --- | --- | --- | --- | --- | --- | --- |
| 98 | 1814 | Alveolata | Dinophyceae | *Adenoides eludens* | KY980192.1 | 99.82 | 1694 |  |  |  |
| 627 | 1816 | Alveolata | Dinophyceae | *Akashiwo sanguinea* | OK638976.1 | 99.94 | 1817 | (Xu et al. 2020) | ● | (Gu et al. 2022) |
| 961 | 1815 | Alveolata | Dinophyceae | *Alexandrium affine* | AY775286.1 | 99.89 | 1789 |  | ● | (Nakanishi et al. 1996) |
| 669 | 1814 | Alveolata | Dinophyceae | *Alexandrium hiranoi* | AY641564.1 | 99.83 | 1799 |  | ● | (Harris et al. 2020) |
| 813 | 1810 | Alveolata | Dinophyceae | *Alexandrium ostenfeldii* | KJ362003.1 | 100.00 | 1669 |  | ● | (Gu et al. 2022) |
| 169 | 1817 | Alveolata | Dinophyceae | *Amoebophrya* sp. *ex Alexandrium affine* | AY775284.1 | 97.88 | 1790 |  |  |  |
| 107 | 1814 | Alveolata | Dinophyceae | *Amoebophrya* sp. *ex Margalefidinium polykrikoides* | KF791347.1 | 98.50 | 1666 |  |  |  |
| 97 | 1809 | Alveolata | Dinophyceae | *Amoebophrya* sp. *ex Margalefidinium polykrikoides* | KF791347.1 | 99.34 | 1666 |  |  |  |
| 883 | 1816 | Alveolata | Dinophyceae | *Amoebophrya* sp. *ex Margalefidinium polykrikoides* | KF791348.1 | 98.28 | 1619 |  |  |  |
| 329 | 1808 | Alveolata | Dinophyceae | *Amoebophrya* sp. *ex Ostreopsis lenticularis* | MW363873.1 | 99.20 | 2340 |  |  |  |
| 541 | 1814 | Alveolata | Dinophyceae | *Amoebophrya* sp. *'Dinophysis'* | AF239260.1 | 97.53 | 1699 |  |  |  |
| 319 | 1814 | Alveolata | Dinophyceae | *Biecheleria brevisulcata* | LC068842.1 | 99.50 | 5637 |  |  |  |
| 161 | 1814 | Alveolata | Dinophyceae | *Ceratium furca* | AJ276699.1 | 96.99 | 1841 | (Chen et al. 2010) | ● | (Burkholder et al. 2008) |
| 1034 | 1812 | Alveolata | Dinophyceae | *Chytriodinium* sp. *Atlantic* | KM245128.1 | 99.83 | 3234 |  |  |  |
| 979 | 1806 | Alveolata | Dinophyceae | *Margalefidinium polykrikoides* | KJ561350.1 | 99.22 | 4028 |  | ● | (Burkholder et al. 2008) |
| 1357 | 1814 | Alveolata | Dinophyceae | *Margalefidinium polykrikoides* | AY347309.2 | 97.61 | 6823 |  | ● | (Burkholder et al. 2008) |
| 880 | 1812 | Alveolata | Dinophyceae | *Dinophysis rotundata* | AJ506975.1 | 98.17 | 1801 |  | ● | (Frehi et al. 2007) |
| 989 | 1796 | Alveolata | Dinophyceae | *Euduboscquella crenulata* | JN606065.1 | 99.33 | 3668 |  |  |  |
| 1266 | 1807 | Alveolata | Dinophyceae | *Fragilidium* sp. | EF492489.1 | 98.10 | 1787 |  |  |  |
| 359 | 1812 | Alveolata | Dinophyceae | *Gonyaulax polygramma* | AJ833631.1 | 99.39 | 1793 | (Chen et al. 2010) | ● | (Burkholder et al. 2008) |
| 423 | 1815 | Alveolata | Dinophyceae | *Gonyaulax polygramma* | AJ833631.1 | 98.56 | 1793 | (Chen et al. 2010) | ● | (Burkholder et al. 2008) |
| 13 | 1815 | Alveolata | Dinophyceae | *Gymnodinium aureolum* | DQ779991.1 | 98.67 | 5897 |  | ● | (Johnsen and Sakshaug 1993) |
| 437 | 1813 | Alveolata | Dinophyceae | *Gymnodinium aureolum* | DQ779991.1 | 97.33 | 5897 |  | ● | (Johnsen and Sakshaug 1993) |
| 507 | 1815 | Alveolata | Dinophyceae | *Gymnodinium aureolum* | DQ779991.1 | 99.61 | 5897 |  | ● | (Johnsen and Sakshaug 1993) |
| 1451 | 1813 | Alveolata | Dinophyceae | *Gymnodinium aureolum* | DQ779991.1 | 97.83 | 5897 |  | ● | (Johnsen and Sakshaug 1993) |
| 203 | 1819 | Alveolata | Dinophyceae | *Gymnodinium aureolum* | DQ779991.1 | 97.12 | 5897 |  | ● | (Johnsen and Sakshaug 1993) |
| 367 | 1812 | Alveolata | Dinophyceae | *Gymnodinium beii* | U37365.1 | 99.94 | 1799 |  |  |  |
| 681 | 1814 | Alveolata | Dinophyceae | *Gymnodinium cf. nolleri* | AB265963.1 | 99.66 | 1755 |  |  |  |
| 152 | 1819 | Alveolata | Dinophyceae | *Gymnodinium heterostriatum* | MT677910.2 | 98.80 | 1744 |  |  |  |
| 425 | 1813 | Alveolata | Dinophyceae | *Gymnodinium* sp. | AF022196.1 | 97.00 | 1803 | (Lai et al. 2017) |  |  |
| 474 | 1818 | Alveolata | Dinophyceae | *Gymnodinium* sp. | AF022196.1 | 97.78 | 1803 | (Lai et al. 2017) |  |  |
| 99 | 1813 | Alveolata | Dinophyceae | *Gyrodinium cf. gutrula* | FN669511.1 | 99.83 | 1796 |  |  |  |
| 1412 | 1812 | Alveolata | Dinophyceae | *Gyrodinium jinhaense* | MK007562.1 | 98.09 | 3232 |  |  |  |
| 1237 | 1821 | Alveolata | Dinophyceae | *Heterocapsa* sp. | FJ549370.1 | 99.22 | 1796 |  |  |  |
| 430 | 1813 | Alveolata | Dinophyceae | *Karlodinium micrum* | JF791048.1 | 97.83 | 1777 |  | ● | (Gu et al. 2022) |
| 1291 | 1815 | Alveolata | Dinophyceae | *Karlodinium micrum* | EF492506.1 | 99.94 | 1794 |  | ● | (Gu et al. 2022) |
| 17 | 1807 | Alveolata | Dinophyceae | *Lepidodinium viride* | AF022199.1 | 99.00 | 1803 |  |  |  |
| 14 | 1799 | Alveolata | Dinophyceae | *Noctiluca scintillans* | AF022200.1 | 97.98 | 1787 | (Lai et al. 2017) | ● | (Gu et al. 2022) |
| 1347 | 1814 | Alveolata | Dinophyceae | *Oodinium pouchetii* | KM879219.1 | 100.00 | 1730 |  |  |  |
| 351 | 1814 | Alveolata | Dinophyceae | *Paragymnodinium* sp. | LC516501.1 | 99.10 | 1770 |  |  |  |
| 1446 | 1815 | Alveolata | Dinophyceae | *Pentapharsodinium tyrrhenicum* | AF022201.1 | 98.44 | 1803 |  |  |  |
| 808 | 1815 | Alveolata | Dinophyceae | *Pentapharsodinium tyrrhenicum* | AF022201.1 | 99.06 | 1803 |  |  |  |
| 691 | 1868 | Alveolata | Dinophyceae | *Pentapharsodinium tyrrhenicum* | AF022201.1 | 99.06 | 1803 |  |  |  |
| 160 | 1814 | Alveolata | Dinophyceae | *Prorocentrum micans* | MK405477.1 | 97.89 | 3848 | (Lai et al. 2017) | ● | (Burkholder et al. 2008) |
| 909 | 1816 | Alveolata | Dinophyceae | *Polykrikos kofoidii* | DQ371291.1 | 99.72 | 1754 |  |  |  |
| 561 | 1815 | Alveolata | Dinophyceae | *Polykrikos kofoidii* | DQ371291.1 | 99.60 | 1754 |  |  |  |
| 1354 | 1814 | Alveolata | Dinophyceae | *Polykrikos* sp. | KY129816.1 | 99.32 | 3151 |  |  |  |
| 556 | 1810 | Alveolata | Dinophyceae | *Protoperidinium bipes* | AB284159.1 | 98.87 | 1765 |  |  |  |
| 1197 | 1841 | Alveolata | Dinophyceae | *Protoperidinium divergens* | AB181892.1 | 99.65 | 1738 |  | ● | (Guo et al. 2004) |
| 501 | 1807 | Alveolata | Dinophyceae | *Protoperidinium pellucidum* | AB181902.1 | 99.89 | 1741 |  | ● | (Guo et al. 2004) |
| 298 | 1814 | Alveolata | Dinophyceae | *Scrippsiella trochoidea* | KR535601.1 | 98.11 | 1799 | (Chen et al. 2010) | ● | (Burkholder et al. 2008) |
| 1037 | 1807 | Alveolata | Dinophyceae | *Syndinium turbo* | DQ146404.1 | 99.89 | 2409 |  |  |  |
| 259 | 1811 | Alveolata | Dinophyceae | *Torodinium teredo* | KR139783.1 | 99.47 | 1700 |  |  |  |
| 309 | 1814 | Alveolata | Dinophyceae | *Warnowia* sp. | FJ947046.1 | 98.65 | 1789 |  |  |  |
| 791 | 1869 | Alveolata | Dinophyceae | *Warnowia* sp. | FJ947040.1 | 98.59 | 1779 |  |  |  |
| 795 | 1813 | Ochrophyta | Bacillariophyceae | *Actinocyclus curvatulus* | X85401.2 | 97.78 | 1800 |  |  |  |
| 1055 | 1801 | Ochrophyta | Bacillariophyceae | *Asterionellopsis glacialis* | AY216904.1 | 99.83 | 1796 | (Xu et al. 2022) | ● | (Guo et al. 2004) |
| 1092 | 1825 | Ochrophyta | Bacillariophyceae | *Asteromphalus* sp. | KJ577845.1 | 99.33 | 1641 |  |  |  |
| 685 | 1800 | Ochrophyta | Bacillariophyceae | *Bacteriastrum mediterraneum* | MG972207.1 | 97.68 | 1765 |  |  |  |
| 196 | 1812 | Ochrophyta | Bacillariophyceae | *Cerataulina pelagica* | HQ912669.1 | 99.76 | 1636 |  |  |  |
| 251 | 1812 | Ochrophyta | Bacillariophyceae | *Cerataulina pelagica* | HQ912669.1 | 99.82 | 1636 |  |  |  |
| 320 | 1810 | Ochrophyta | Bacillariophyceae | *Chaetoceros socialis* | KY852276.1 | 99.94 | 1796 |  | ● | (Tomaru et al. 2009) |
| 668 | 1811 | Ochrophyta | Bacillariophyceae | *Chaetoceros eibenii* | MG972280.1 | 99.47 | 1710 | (Chen et al. 2010) | ● | (Jiao et al. 2010) |
| 714 | 1807 | Ochrophyta | Bacillariophyceae | *Chaetoceros contortus* | MG972222.1 | 99.18 | 1713 |  | ● | (Shevchenko and Orlova 2010) |
| 626 | 1802 | Ochrophyta | Bacillariophyceae | *Chaetoceros pseudocurvisetus* | MW832834.1 | 99.83 | 1742 | (Wang et al. 2015) | ● | (Guo et al. 2004) |
| 296 | 1808 | Ochrophyta | Bacillariophyceae | *Chaetoceros elegans* | KX611421.1 | 99.75 | 1596 |  |  |  |
| 908 | 1808 | Ochrophyta | Bacillariophyceae | *Chaetoceros cf. lauderi* | MK795712.1 | 99.37 | 1742 |  | ● | (Jiao et al. 2010) |
| 1399 | 1794 | Ochrophyta | Bacillariophyceae | *Chaetoceros* sp. | AB847416.1 | 99.71 | 1747 |  |  |  |
| 1438 | 1821 | Ochrophyta | Bacillariophyceae | *Coscinodiscus jonesianus* | KJ577852.1 | 99.32 | 1773 | (Chen et al. 2010) | ● | (Guo et al. 2004) |
| 713 | 1813 | Ochrophyta | Bacillariophyceae | *Cyclotella choctawhatcheeana* | AM712618.1 | 99.17 | 1803 |  |  |  |
| 191 | 1798 | Ochrophyta | Bacillariophyceae | *Cylindrotheca closterium* | KY045848.1 | 98.77 | 1701 |  | ● | (Wang 2002) |
| 876 | 1814 | Ochrophyta | Bacillariophyceae | *Fragilariopsis kerguelensis* | LR812489.1 | 97.17 | 10223 |  |  |  |
| 42 | 1813 | Ochrophyta | Bacillariophyceae | *Guinardia striata* | MW750344.1 | 99.77 | 1753 | (Xu et al. 2022) | ● | (Guo et al. 2004) |
| 564 | 1815 | Ochrophyta | Bacillariophyceae | *Hemiaulus sinensis* | HQ912624.1 | 99.76 | 1650 | (Xu et al. 2022) |  |  |
| 153 | 1818 | Ochrophyta | Bacillariophyceae | *Leptocylindrus convexus* | KC814811.1 | 99.77 | 1731 |  |  |  |
| 104 | 1817 | Ochrophyta | Bacillariophyceae | *Leptocylindrus danicus* | AJ535175.1 | 99.56 | 1649 | (Wang et al. 2015) | ● | (Guo et al. 2004) |
| 123 | 1815 | Ochrophyta | Bacillariophyceae | *Minidiscus trioculatus* | DQ093363.1 | 98.51 | 1805 |  |  |  |
| 473 | 1822 | Ochrophyta | Bacillariophyceae | *Minutocellus polymorphus* | HQ912568.1 | 99.83 | 1793 |  |  |  |
| 548 | 1804 | Ochrophyta | Bacillariophyceae | *Navicula arenaria* | KJ961668.1 | 99.16 | 1789 |  |  |  |
| 768 | 1802 | Ochrophyta | Bacillariophyceae | *Navicula arenaria* | KJ961668.1 | 98.49 | 1789 |  |  |  |
| 924 | 1807 | Ochrophyta | Bacillariophyceae | *Pleurosigma* sp. | KJ961710.1 | 97.93 | 1789 | (Chen et al. 2010) |  |  |
| 903 | 1814 | Ochrophyta | Bacillariophyceae | *Proboscia indica* | AY485470.1 | 99.52 | 1660 |  |  |  |
| 915 | 1806 | Ochrophyta | Bacillariophyceae | *Paraphysomonas imperforata* | EF432518.1 | 99.61 | 1793 |  |  |  |
| 34 | 1814 | Ochrophyta | Bacillariophyceae | *Pseudo-nitzschia americana* | MZ267110.2 | 99.11 | 1788 | (Xu et al. 2022) |  |  |
| 46 | 1803 | Ochrophyta | Bacillariophyceae | *Pseudo-nitzschia micropora* | KP709003.1 | 99.75 | 1628 |  |  |  |
| 218 | 1817 | Ochrophyta | Bacillariophyceae | *Rhizosolenia fallax* | AY485480.1 | 100.00 | 1703 |  |  |  |
| 1138 | 1813 | Ochrophyta | Bacillariophyceae | *Rhizosolenia setigera* | MW344861.1 | 100.00 | 1775 | (Chen et al. 2010) | ● | (Guo et al. 2004) |
| 836 | 1815 | Ochrophyta | Bacillariophyceae | *Rhizosolenia imbricate* | KC309543.1 | 99.78 | 1340 |  | ● | (Jiao et al. 2010) |
| 387 | 1811 | Ochrophyta | Bacillariophyceae | *Skeletonema menzellii* | DQ011161.1 | 99.08 | 1730 |  |  |  |
| 18 | 1812 | Ochrophyta | Bacillariophyceae | *Skeletonema* sp. | MT499904.1 | 100.00 | 1794 |  |  |  |
| 1162 | 1818 | Ochrophyta | Bacillariophyceae | *Skeletonema* sp. | MT499904.1 | 99.67 | 1794 |  |  |  |
| 15 | 1814 | Ochrophyta | Bacillariophyceae | *Skeletonema* sp. | MT499904.1 | 99.89 | 1794 |  |  |  |
| 1423 | 1817 | Ochrophyta | Bacillariophyceae | *Thalassiosira hendeyi* | AM050629.1 | 97.78 | 1803 |  |  |  |
| 168 | 1813 | Ochrophyta | Bacillariophyceae | *Thalassiosira mala* | HM991693.1 | 98.75 | 1758 |  | ● | (Liu et al. 2014) |
| 85 | 1814 | Ochrophyta | Bacillariophyceae | *Thalassiosira* sp. | MT489371.1 | 99.94 | 1676 | (Wang et al. 2015) |  |  |
| 75 | 1812 | Ochrophyta | Bacillariophyceae | *Thalassiosira* sp. | MT489371.1 | 98.88 | 1676 | (Wang et al. 2015) |  |  |
| 1259 | 1801 | Ochrophyta | Bacillariophyceae | *Thalassiothrix longissima* | AB430611.1 | 98.20 | 1773 | (Chen et al. 2010) |  |  |
| 779 | 1790 | Chlorophyta | Prasinophyceae | *Bathycoccus prasinos* | XR_002608757.1 | 99.89 | 1775 |  |  |  |
| 1262 | 1803 | Chlorophyta | Prasinophyceae | *Crustomastix stigmatica* | KY980091.1 | 98.10 | 1691 |  |  |  |
| 1172 | 1805 | Chlorophyta | Prasinophyceae | *Dolichomastix tenuilepis* | FN562449.1 | 97.07 | 2438 |  |  |  |
| 339 | 1795 | Chlorophyta | Prasinophyceae | *Mamiella gilva* | FN562450.1 | 99.55 | 2362 |  |  |  |
| 1383 | 1793 | Chlorophyta | Prasinophyceae | *Micromonas bravo* | MT117944.1 | 98.26 | 5525 |  |  |  |
| 1283 | 1793 | Chlorophyta | Prasinophyceae | *Micromonas bravo* | MT117944.1 | 98.26 | 5525 |  |  |  |
| 12 | 1906 | Chlorophyta | Prasinophyceae | *Micromonas bravo* | MT117944.1 | 99.94 | 5525 |  |  |  |
| 16 | 1793 | Chlorophyta | Prasinophyceae | *Micromonas* sp. | HM191693.1 | 100.00 | 1777 |  |  |  |
| 1098 | 1842 | Chlorophyta | Prasinophyceae | *Nephroselmis pyriformis* | AY425306.1 | 99.83 | 1758 |  |  |  |
| 78 | 1782 | Chlorophyta | Prasinophyceae | *Ostreococcus* sp. *'lucimarinus'* | MT117941.1 | 99.09 | 5509 |  |  |  |
| 64 | 1910 | Chlorophyta | Prasinophyceae | *Ostreococcus* sp. *'lucimarinus'* | MT117941.1 | 98.54 | 5509 |  |  |  |
| 1026 | 1814 | Chlorophyta | Prasinophyceae | *Prasinoderma coloniale* | KT860886.1 | 96.06 | 1631 |  |  |  |
| 1101 | 1808 | Chlorophyta | Prasinophyceae | *Pterosperma* sp. | KF899835.1 | 98.67 | 1659 |  |  |  |
| 1072 | 1817 | Chlorophyta | Prasinophyceae | *Pterosperma* sp. | KF899835.1 | 98.98 | 1659 |  |  |  |
| 744 | 1802 | Chlorophyta | Prasinophyceae | *Pycnococcus provasolii* | X91264.1 | 99.67 | 1793 |  |  |  |
| 537 | 1807 | Chlorophyta | Prasinophyceae | *Pycnococcus provasolii* | X91264.1 | 99.61 | 1793 |  |  |  |
| 389 | 1819 | Chlorophyta | Prasinophyceae | *Pyramimonas obovata* | KF422615.1 | 99.71 | 1695 |  |  |  |
| 414 | 1816 | Chlorophyta | Prasinophyceae | *Pyramimonas parkeae* | KX611141.1 | 98.50 | 1802 |  |  |  |
| 569 | 1811 | Chlorophyta | Prasinophyceae | *Tetraselmis* sp. | MG022697.1 | 97.16 | 2322 |  |  |  |
| 121 | 1808 | Haptophyta | Prymnesiophyceae | *Chrysochromulina campanulifera* | AJ246273.1 | 98.67 | 1799 |  |  |  |
| 232 | 1812 | Haptophyta | Prymnesiophyceae | *Chrysochromulina cymbium* | AM491018.1 | 98.86 | 1787 |  |  |  |
| 108 | 1814 | Haptophyta | Prymnesiophyceae | *Chrysochromulina leadbeateri* | AM491017.2 | 98.32 | 1785 |  | ● | (Burkholder et al. 2008) |
| 1224 | 1814 | Haptophyta | Prymnesiophyceae | *Chrysochromulina scutellum* | AJ246274.1 | 98.04 | 1786 |  |  |  |
| 1184 | 1810 | Haptophyta | Prymnesiophyceae | *Chrysochromulina scutellum* | AJ246274.1 | 97.43 | 1786 |  |  |  |
| 1240 | 1813 | Haptophyta | Prymnesiophyceae | *Chrysochromulina scutellum* | AJ246274.1 | 97.26 | 1786 |  |  |  |
| 1227 | 1813 | Haptophyta | Prymnesiophyceae | *Gephyrocapsa oceanica* | AB058360.1 | 99.94 | 1755 |  | ● | (Rhodes et al. 1995) |
| 1281 | 1831 | Haptophyta | Prymnesiophyceae | *Haptophyceae* sp. | LC599498.1 | 99.89 | 1771 |  |  |  |
| 1378 | 1832 | Haptophyta | Prymnesiophyceae | *Haptophyceae* sp. | LC599498.1 | 97.69 | 1771 |  |  |  |
| 237 | 1815 | Haptophyta | Prymnesiophyceae | *Haptolina fragaria* | AM491013.2 | 98.83 | 1789 |  |  |  |
| 680 | 1816 | Haptophyta | Prymnesiophyceae | *Helicosphaera carteri* | AM490983.2 | 98.61 | 1724 |  | ● | (Moita et al. 2010) |
| 1430 | 1814 | Haptophyta | Prymnesiophyceae | *Helicosphaera carteri* | AM490983.2 | 98.55 | 1724 |  | ● | (Moita et al. 2010) |
| 53 | 1816 | Haptophyta | Prymnesiophyceae | *Phaeocystis cordata* | JX660992.1 | 99.88 | 1761 |  | ● | (Zingone et al. 1999) |
| 310 | 1814 | Haptophyta | Prymnesiophyceae | *Phaeocystis jahnii* | LC189148.1 | 97.32 | 1714 |  | ● | (Zingone et al. 1999) |
| 90 | 1787 | Cryptophyceae | Cryptophyceae | *Geminigera cryophila* | DQ452091.1 | 98.59 | 1770 |  |  |  |
| 1431 | 1850 | Cryptophyceae | Cryptophyceae | *Hemiselmis aquamarina* | MF179483.1 | 100.00 | 1811 |  |  |  |
| 1068 | 1790 | Cryptophyceae | Cryptophyceae | *Hemiselmis andersenii* | MF179473.1 | 99.55 | 1844 |  |  |  |
| 1195 | 1798 | Cryptophyceae | Cryptophyceae | *Hemiarma marina* | LC151286.1 | 99.94 | 1655 |  |  |  |
| 393 | 1869 | Cryptophyceae | Cryptophyceae | *Leucocryptos marina* | DQ980481.1 | 99.77 | 1731 |  |  |  |
| 484 | 1784 | Cryptophyceae | Cryptophyceae | *Rhodomonas* sp. | AJ421148.1 | 99.77 | 1707 |  |  |  |
| 428 | 1863 | Cryptophyceae | Cryptophyceae | *Teleaulax amphioxeia* | AJ421146.1 | 99.94 | 1787 |  | ● | (Du Yoo et al. 2017) |
| 76 | 1775 | Cryptophyceae | Cryptophyceae | *Teleaulax amphioxeia* | KY980412.1 | 99.64 | 1668 |  | ● | (Du Yoo et al. 2017) |
| 1166 | 1790 | Cryptophyceae | Cryptophyceae | *Proteomonas* sp. | MK828429.1 | 98.89 | 1712 |  |  |  |
| 1017 | 1804 | Ochrophyta | Chrysophyceae | *Chrysoxys* sp. | AF123302.1 | 98.10 | 1790 |  |  |  |
| 1229 | 1803 | Ochrophyta | Chrysophyceae | *Paraphysomonas butcheri* | AF109326.1 | 98.59 | 1707 |  |  |  |
| 1436 | 1822 | Ochrophyta | Chrysophyceae | *Pedinellales* sp. | JN934682.1 | 98.62 | 1665 |  |  |  |
| 1441 | 1835 | Ochrophyta | Raphidophyceae | *Chattonella marina* | AB217626.1 | 100.00 | 1818 | (Xu et al. 2021) | ● | (Gu et al. 2022) |
| 297 | 1853 | Ochrophyta | Raphidophyceae | *Fibrocapsa japonica* | AY788931.1 | 100.00 | 1798 |  | ● | (Cucchiari et al. 2010) |
| 636 | 1827 | Ochrophyta | Dictyochophyceae | *Florenciella* sp. | KT861110.1 | 100.00 | 1658 |  |  |  |
| 496 | 1827 | Ochrophyta | Dictyochophyceae | *Pseudochattonella verruculosa* | AY788948.1 | 99.83 | 1814 |  | ● | (MacKenzie et al. 2011) |
| 1385 | 1825 | Ochrophyta | Pinguiophyceae | *Phaeomonas parva* | AB042204.1 | 99.39 | 1811 |  |  |  |

**Supplementary table 2** Phytoplankton genera and OTU number identified by short-read metabarcoding

| **Taxon** | **2016/09** | **2016/11** | **2016/12** | **2017/01** | **2017/02** | **2017/03** | **2017/06** | **2017/08** |
| --- | --- | --- | --- | --- | --- | --- | --- | --- |
| k__Eukaryota;p__unidentified;c__Dinophyceae;o__Gymnodiniales;f__Kareniaceae;g__Karlodinium | 3043 | 2857 | 8871 | 5005 | 613 | 898 | 10354 | 6681 |
| k__Eukaryota;p__unidentified;c__Dinophyceae;o__Syndiniales;f__Amoebophryaceae;g__Amoebophrya | 2056 | 1004 | 4699 | 4384 | 2021 | 2538 | 1316 | 5545 |
| k__Eukaryota;p__unidentified;c__unidentified;o__Phaeocystales;f__Phaeocystaceae;g__Phaeocystis | 368 | 112 | 4975 | 1610 | 2038 | 5624 | 809 | 117 |
| k__Eukaryota;p__unidentified;c__Dinophyceae;o__Noctilucales;f__Noctilucaceae;g__Noctiluca | 69 | 14 | 0 | 119 | 4388 | 7041 | 0 | 4 |
| k__Eukaryota;p__unidentified;c__Dinophyceae;o__Gymnodiniales;f__Gymnodiniaceae;g__Lepidodinium | 1912 | 461 | 775 | 1318 | 804 | 1893 | 2166 | 1269 |
| k__Eukaryota;p__unidentified;c__Dinophyceae;o__Gymnodiniales;f__Kareniaceae;g__Karenia | 3235 | 1191 | 122 | 1022 | 1558 | 1042 | 59 | 1313 |
| k__Eukaryota;p__unidentified;c__Dinophyceae;o__Prorocentrales;f__Prorocentraceae;g__Prorocentrum | 954 | 755 | 1777 | 610 | 850 | 880 | 511 | 890 |
| k__Eukaryota;p__unidentified;c__unidentified;o__Prymnesiales;f__Chrysochromulinaceae;g__Chrysochromulina | 1133 | 2548 | 255 | 148 | 464 | 997 | 407 | 891 |
| k__Eukaryota;p__Chlorophyta;c__Mamiellophyceae;o__Mamiellales;f__Bathycoccaceae;g__Ostreococcus | 16 | 8 | 19 | 0 | 18 | 2387 | 1153 | 0 |
| k__Eukaryota;p__Bacillariophyta;c__Coscinodiscophyceae;o__Chaetocerotales;f__Chaetocerotaceae;g__Chaetoceros | 244 | 8 | 16 | 513 | 2655 | 50 | 17 | 32 |
| k__Eukaryota;p__unidentified;c__unidentified;o__Pyrenomonadales;f__Geminigeraceae;g__Teleaulax | 32 | 904 | 810 | 297 | 20 | 221 | 686 | 3 |
| k__Eukaryota;p__unidentified;c__Dinophyceae;o__Gymnodiniales;f__Kareniaceae;g__Takayama | 9 | 22 | 2623 | 0 | 0 | 0 | 56 | 15 |
| k__Eukaryota;p__unidentified;c__unidentified;o__Syracosphaerales;f__Syracosphaeraceae;g__Syracosphaera | 396 | 501 | 76 | 23 | 38 | 423 | 168 | 431 |
| k__Eukaryota;p__unidentified;c__Dinophyceae;o__Gymnodiniales;f__Brachidiniaceae;g__Brachidinium | 480 | 63 | 33 | 35 | 2 | 19 | 356 | 1008 |
| k__Eukaryota;p__unidentified;c__Dinophyceae;o__Gymnodiniales;f__Gymnodiniaceae;g__Gymnodinium | 749 | 21 | 117 | 132 | 100 | 175 | 32 | 455 |
| k__Eukaryota;p__Chlorophyta;c__Mamiellophyceae;o__Mamiellales;f__Mamiellaceae;g__Micromonas | 12 | 959 | 121 | 0 | 49 | 86 | 295 | 0 |
| k__Eukaryota;p__unidentified;c__Dinophyceae;o__Peridiniales;f__Thoracosphaeraceae;g__Scrippsiella | 312 | 287 | 19 | 141 | 244 | 223 | 36 | 255 |
| k__Eukaryota;p__unidentified;c__Dinophyceae;o__Peridiniales;f__unidentified;g__Peridiniopsis | 132 | 4 | 14 | 285 | 151 | 8 | 779 | 140 |
| k__Eukaryota;p__Bacillariophyta;c__Coscinodiscophyceae;o__Leptocylindrales;f__Leptocylindraceae;g__Leptocylindrus | 1144 | 0 | 0 | 0 | 0 | 0 | 0 | 171 |
| k__Eukaryota;p__unidentified;c__Dinophyceae;o__Gymnodiniales;f__Warnowiaceae;g__Warnowia | 99 | 0 | 85 | 280 | 575 | 130 | 42 | 93 |
| k__Eukaryota;p__unidentified;c__Dinophyceae;o__Peridiniales;f__Peridiniaceae;g__Pentapharsodinium | 331 | 22 | 138 | 88 | 23 | 83 | 137 | 450 |
| k__Eukaryota;p__unidentified;c__Dinophyceae;o__Gymnodiniales;f__Gymnodiniaceae;g__Gyrodinium | 322 | 130 | 9 | 251 | 238 | 3 | 11 | 281 |
| k__Eukaryota;p__unidentified;c__Dinophyceae;o__Blastodiniales;f__unidentified;g__Blastodinium | 114 | 10 | 132 | 679 | 134 | 21 | 2 | 21 |
| k__Eukaryota;p__unidentified;c__Dinophyceae;o__Suessiales;f__Suessiaceae;g__Pelagodinium | 21 | 267 | 7 | 239 | 342 | 32 | 1 | 38 |
| k__Eukaryota;p__unidentified;c__Dinophyceae;o__Suessiales;f__Suessiaceae;g__Polarella | 0 | 0 | 902 | 1 | 5 | 17 | 0 | 15 |
| k__Eukaryota;p__unidentified;c__Dinophyceae;o__Torodiniales;f__Torodiniaceae;g__Torodinium | 245 | 20 | 42 | 109 | 255 | 78 | 47 | 96 |
| k__Eukaryota;p__unidentified;c__unidentified;o__Prymnesiales;f__Prymnesiaceae;g__Haptolina | 311 | 233 | 4 | 8 | 21 | 13 | 144 | 67 |
| k__Eukaryota;p__unidentified;c__Dinophyceae;o__Peridiniales;f__Podolampaceae;g__Podolampas | 275 | 6 | 0 | 15 | 242 | 48 | 166 | 22 |
| k__Eukaryota;p__Bacillariophyta;c__Coscinodiscophyceae;o__Rhizosoleniales;f__Rhizosoleniaceae;g__Rhizosolenia | 258 | 0 | 0 | 62 | 32 | 0 | 0 | 335 |
| k__Eukaryota;p__Bacillariophyta;c__Mediophyceae;o__Hemiaulales;f__Hemiaulaceae;g__Eucampia | 0 | 0 | 0 | 87 | 595 | 0 | 0 | 0 |
| k__Eukaryota;p__unidentified;c__Dinophyceae;o__Peridiniales;f__Endodiniaceae;g__Brandtodinium | 23 | 49 | 17 | 18 | 3 | 2 | 539 | 17 |
| k__Eukaryota;p__unidentified;c__unidentified;o__unidentified;f__unidentified;g__Gladiolithus | 53 | 108 | 245 | 15 | 31 | 115 | 9 | 22 |
| k__Eukaryota;p__unidentified;c__Dinophyceae;o__Suessiales;f__Biecheleriaceae;g__Biecheleria | 189 | 246 | 0 | 14 | 0 | 37 | 0 | 86 |
| k__Eukaryota;p__unidentified;c__Dinophyceae;o__unidentified;f__unidentified;g__Pseudadenoides | 435 | 35 | 0 | 5 | 4 | 2 | 1 | 34 |
| k__Eukaryota;p__Bacillariophyta;c__Coscinodiscophyceae;o__Thalassiosirales;f__Thalassiosiraceae;g__Thalassiosira | 0 | 441 | 6 | 5 | 40 | 0 | 0 | 5 |
| k__Eukaryota;p__unidentified;c__unidentified;o__Pyrenomonadales;f__Geminigeraceae;g__Geminigera | 0 | 348 | 131 | 3 | 7 | 0 | 1 | 0 |
| k__Eukaryota;p__unidentified;c__unidentified;o__Prymnesiales;f__Prymnesiaceae;g__Prymnesium | 211 | 0 | 31 | 15 | 8 | 17 | 102 | 88 |
| k__Eukaryota;p__unidentified;c__unidentified;o__unidentified;f__unidentified;g__Ichthyodinium | 133 | 87 | 2 | 69 | 25 | 25 | 1 | 91 |
| k__Eukaryota;p__Bacillariophyta;c__Bacillariophyceae;o__Bacillariales;f__Bacillariaceae;g__Pseudo-nitzschia | 11 | 0 | 0 | 114 | 178 | 0 | 1 | 84 |
| k__Eukaryota;p__unidentified;c__Dinophyceae;o__Peridiniales;f__Pfiesteriaceae;g__Pfiesteria | 187 | 0 | 4 | 0 | 87 | 0 | 0 | 75 |
| k__Eukaryota;p__unidentified;c__Dinophyceae;o__Gymnodiniales;f__Gymnodiniaceae;g__Nusuttodinium | 0 | 204 | 10 | 64 | 10 | 10 | 15 | 33 |
| k__Eukaryota;p__Bacillariophyta;c__Coscinodiscophyceae;o__Rhizosoleniales;f__Rhizosoleniaceae;g__Guinardia | 57 | 0 | 0 | 0 | 180 | 0 | 0 | 103 |
| k__Eukaryota;p__unidentified;c__unidentified;o__Coccosphaerales;f__unidentified;g__Pontosphaeraceae | 30 | 21 | 179 | 0 | 0 | 68 | 22 | 5 |
| k__Eukaryota;p__unidentified;c__Chrysophyceae;o__Chromulinales;f__Chromulinaceae;g__Ochromonas | 0 | 320 | 0 | 0 | 0 | 1 | 0 | 0 |
| k__Eukaryota;p__unidentified;c__unidentified;o__unidentified;f__unidentified;g__Telonema | 50 | 211 | 0 | 5 | 9 | 1 | 0 | 32 |
| k__Eukaryota;p__Bacillariophyta;c__Mediophyceae;o__Hemiaulales;f__Hemiaulaceae;g__Hemiaulus | 29 | 0 | 23 | 150 | 95 | 0 | 3 | 2 |
| k__Eukaryota;p__unidentified;c__Dinophyceae;o__Gonyaulacales;f__Goniodomataceae;g__Gambierdiscus | 0 | 0 | 65 | 223 | 0 | 0 | 0 | 0 |
| k__Eukaryota;p__unidentified;c__unidentified;o__Coccolithales;f__Hymenomonadaceae;g__Ochrosphaera | 37 | 0 | 0 | 0 | 0 | 209 | 8 | 26 |
| k__Eukaryota;p__Eustigmatophyceae;c__unidentified;o__Eustigmatales;f__Monodopsidaceae;g__Nannochloropsis | 132 | 0 | 123 | 0 | 0 | 0 | 0 | 0 |
| k__Eukaryota;p__unidentified;c__Dinophyceae;o__Peridiniales;f__Heterocapsaceae;g__Heterocapsa | 36 | 2 | 153 | 4 | 35 | 5 | 2 | 15 |
| k__Eukaryota;p__Chlorophyta;c__unidentified;o__unidentified;f__Pycnococcaceae;g__Pycnococcus | 145 | 20 | 0 | 0 | 0 | 0 | 0 | 39 |
| k__Eukaryota;p__unidentified;c__Dinophyceae;o__Dinophysiales;f__unidentified;g__Triposolenia | 26 | 3 | 9 | 44 | 14 | 17 | 0 | 57 |
| k__Eukaryota;p__Bacillariophyta;c__Coscinodiscophyceae;o__Coscinodiscales;f__Heliopeltaceae;g__Actinoptychus | 0 | 0 | 0 | 0 | 168 | 0 | 0 | 0 |
| k__Eukaryota;p__unidentified;c__unidentified;o__Isochrysidales;f__Isochrysidaceae;g__Isochrysis | 0 | 0 | 168 | 0 | 0 | 0 | 0 | 0 |
| k__Eukaryota;p__Chlorophyta;c__Mamiellophyceae;o__Mamiellales;f__Mamiellaceae;g__Mantoniella | 4 | 63 | 36 | 0 | 10 | 3 | 44 | 0 |
| k__Eukaryota;p__Bacillariophyta;c__Mediophyceae;o__Lithodesmiales;f__Lithodesmiaceae;g__Lithodesmium | 0 | 0 | 0 | 0 | 149 | 0 | 0 | 0 |
| k__Eukaryota;p__unidentified;c__unidentified;o__Protosteliales;f__Cavosteliaceae;g__Ceratiomyxella | 0 | 135 | 0 | 0 | 0 | 0 | 0 | 0 |
| k__Eukaryota;p__unidentified;c__unidentified;o__Pyrenomonadales;f__Pyrenomonadaceae;g__Rhodomonas | 0 | 0 | 101 | 8 | 2 | 0 | 16 | 8 |
| k__Eukaryota;p__unidentified;c__Dinophyceae;o__Dinophysiales;f__Dinophysiaceae;g__Sinophysis | 46 | 20 | 3 | 5 | 4 | 32 | 1 | 20 |
| k__Eukaryota;p__Bacillariophyta;c__Mediophyceae;o__Triceratiales;f__Triceratiaceae;g__Trieres | 12 | 0 | 0 | 9 | 5 | 0 | 0 | 99 |
| k__Eukaryota;p__unidentified;c__unidentified;o__Isochrysidales;f__Noelaerhabdaceae;g__Emiliania | 11 | 0 | 0 | 0 | 15 | 55 | 42 | 0 |
| k__Eukaryota;p__unidentified;c__Dinophyceae;o__Gymnodiniales;f__Polykrikaceae;g__Polykrikos | 0 | 0 | 0 | 1 | 105 | 0 | 0 | 0 |
| k__Eukaryota;p__unidentified;c__Dinophyceae;o__unidentified;f__unidentified;g__Stoeckeria | 0 | 0 | 0 | 92 | 12 | 2 | 0 | 0 |
| k__Eukaryota;p__unidentified;c__unidentified;o__Syracosphaerales;f__Rhabdosphaeraceae;g__Algirosphaera | 18 | 0 | 0 | 0 | 0 | 2 | 0 | 86 |
| k__Eukaryota;p__unidentified;c__unidentified;o__unidentified;f__Acanthocystidae;g__Raineriophrys | 36 | 0 | 2 | 3 | 0 | 0 | 40 | 25 |
| k__Eukaryota;p__Chlorophyta;c__unidentified;o__Pyramimonadales;f__unidentified;g__Pyramimonas | 0 | 0 | 5 | 0 | 24 | 0 | 0 | 68 |
| k__Eukaryota;p__unidentified;c__unidentified;o__unidentified;f__unidentified;g__Pirsonia | 21 | 0 | 3 | 10 | 15 | 2 | 0 | 46 |
| k__Eukaryota;p__unidentified;c__Raphidophyceae;o__Chattonellales;f__Chattonellaceae;g__Fibrocapsa | 0 | 0 | 0 | 90 | 0 | 0 | 0 | 0 |
| k__Eukaryota;p__unidentified;c__unidentified;o__unidentified;f__Katablepharidaceae;g__Katablepharis | 13 | 16 | 0 | 19 | 0 | 0 | 0 | 32 |
| k__Eukaryota;p__Chlorophyta;c__Nephroselmidophyceae;o__unidentified;f__unidentified;g__Nephroselmis | 0 | 3 | 2 | 0 | 0 | 0 | 72 | 0 |
| k__Eukaryota;p__unidentified;c__Dinophyceae;o__Gonyaulacales;f__Ceratocoryaceae;g__Ceratocorys | 0 | 0 | 0 | 1 | 0 | 0 | 75 | 0 |
| k__Eukaryota;p__unidentified;c__Dinophyceae;o__Peridiniales;f__Pfiesteriaceae;g__Cryptoperidiniopsis | 0 | 0 | 45 | 27 | 0 | 1 | 0 | 3 |
| k__Eukaryota;p__unidentified;c__Dinophyceae;o__Gymnodiniales;f__Gymnodiniaceae;g__Moestrupia | 4 | 0 | 0 | 0 | 0 | 0 | 0 | 70 |
| k__Eukaryota;p__unidentified;c__Dinophyceae;o__unidentified;f__unidentified;g__Abedinium | 0 | 0 | 0 | 47 | 25 | 0 | 0 | 0 |
| k__Eukaryota;p__unidentified;c__unidentified;o__unidentified;f__Salpingoecidae;g__Bicosta | 0 | 0 | 0 | 0 | 72 | 0 | 0 | 0 |
| k__Eukaryota;p__Chlorophyta;c__Mamiellophyceae;o__Mamiellales;f__Mamiellaceae;g__Mamiella | 63 | 0 | 0 | 0 | 7 | 0 | 0 | 0 |
| k__Eukaryota;p__unidentified;c__Dinophyceae;o__Gonyaulacales;f__Gonyaulacaceae;g__Gonyaulax | 22 | 2 | 0 | 0 | 4 | 0 | 24 | 10 |
| k__Eukaryota;p__unidentified;c__Chrysophyceae;o__Chromulinales;f__Paraphysomonadaceae;g__Paraphysomonas | 6 | 1 | 0 | 26 | 17 | 0 | 11 | 0 |
| k__Eukaryota;p__unidentified;c__Dinophyceae;o__Peridiniales;f__Peridiniaceae;g__Peridinium | 33 | 0 | 0 | 0 | 0 | 13 | 0 | 14 |
| k__Eukaryota;p__unidentified;c__Raphidophyceae;o__Chattonellales;f__Chattonellaceae;g__Heterosigma | 0 | 58 | 2 | 0 | 0 | 0 | 0 | 0 |
| k__Eukaryota;p__unidentified;c__unidentified;o__unidentified;f__unidentified;g__Incisomonas | 3 | 0 | 0 | 0 | 42 | 12 | 0 | 0 |
| k__Eukaryota;p__unidentified;c__Dinophyceae;o__Gymnodiniales;f__Warnowiaceae;g__Proterythropsis | 0 | 0 | 5 | 21 | 7 | 23 | 0 | 0 |
| k__Eukaryota;p__unidentified;c__Dinophyceae;o__Phytodiniales;f__Phytodiniaceae;g__Spiniferodinium | 19 | 0 | 0 | 2 | 29 | 0 | 0 | 4 |
| k__Eukaryota;p__unidentified;c__unidentified;o__unidentified;f__unidentified;g__Cryptocaryon | 0 | 0 | 51 | 0 | 0 | 0 | 2 | 0 |
| k__Eukaryota;p__unidentified;c__Dinophyceae;o__Gymnodiniales;f__Gymnodiniaceae;g__Paulsenella | 45 | 0 | 0 | 0 | 0 | 0 | 0 | 6 |
| k__Eukaryota;p__unidentified;c__unidentified;o__Coccolithales;f__Calcidiscaceae;g__Tergestiella | 46 | 0 | 0 | 0 | 5 | 0 | 0 | 0 |
| k__Eukaryota;p__unidentified;c__unidentified;o__Cryomonadida;f__unidentified;g__Rhogostoma | 0 | 0 | 0 | 2 | 45 | 3 | 0 | 0 |
| k__Eukaryota;p__unidentified;c__unidentified;o__Thaumatomonadida;f__Thaumatomastigidae;g__Thaumatomastix | 4 | 7 | 0 | 3 | 22 | 7 | 2 | 0 |
| k__Eukaryota;p__Bacillariophyta;c__Mediophyceae;o__Biddulphiales;f__Biddulphiaceae;g__Biddulphia | 0 | 0 | 0 | 8 | 36 | 0 | 0 | 0 |
| k__Eukaryota;p__unidentified;c__Dinophyceae;o__Suessiales;f__Suessiaceae;g__Protodinium | 4 | 0 | 0 | 0 | 0 | 27 | 0 | 11 |
| k__Eukaryota;p__unidentified;c__Dinophyceae;o__Pyrocystales;f__Pyrocystaceae;g__Dissodinium | 16 | 0 | 0 | 3 | 21 | 0 | 0 | 0 |
| k__Eukaryota;p__unidentified;c__unidentified;o__unidentified;f__unidentified;g__Minorisa | 0 | 0 | 0 | 7 | 33 | 0 | 0 | 0 |
| k__Eukaryota;p__unidentified;c__Dinophyceae;o__Gymnodiniales;f__Gymnodiniaceae;g__Akashiwo | 21 | 0 | 0 | 8 | 4 | 0 | 0 | 6 |
| k__Eukaryota;p__unidentified;c__unidentified;o__unidentified;f__Apusomonadidae;g__Amastigomonas | 0 | 0 | 0 | 6 | 0 | 33 | 0 | 0 |
| k__Eukaryota;p__unidentified;c__Dinophyceae;o__Gonyaulacales;f__Amphidomataceae;g__Azadinium | 24 | 3 | 0 | 3 | 0 | 0 | 0 | 8 |
| k__Eukaryota;p__Bacillariophyta;c__Coscinodiscophyceae;o__Thalassiosirales;f__Lauderiaceae;g__Lauderia | 0 | 0 | 0 | 27 | 10 | 0 | 0 | 0 |
| k__Eukaryota;p__unidentified;c__Dinophyceae;o__Gymnodiniales;f__Warnowiaceae;g__Nematodinium | 5 | 0 | 0 | 5 | 2 | 18 | 0 | 7 |
| k__Eukaryota;p__unidentified;c__unidentified;o__unidentified;f__Acanthocystidae;g__Pterocystis | 12 | 0 | 0 | 0 | 0 | 0 | 22 | 3 |
| k__Eukaryota;p__unidentified;c__Chrysophyceae;o__Chromulinales;f__Chromulinaceae;g__Spumella | 0 | 0 | 0 | 0 | 3 | 33 | 0 | 0 |
| k__Eukaryota;p__unidentified;c__Dinophyceae;o__Peridiniales;f__Podolampaceae;g__Blepharocysta | 8 | 0 | 0 | 0 | 0 | 0 | 0 | 27 |
| k__Eukaryota;p__unidentified;c__unidentified;o__Pyrenomonadales;f__Chroomonadaceae;g__Chroomonas | 0 | 0 | 35 | 0 | 0 | 0 | 0 | 0 |
| k__Eukaryota;p__unidentified;c__Dinophyceae;o__Coccidiniales;f__Chytriodiniaceae;g__Chytriodinium | 0 | 0 | 6 | 7 | 21 | 0 | 0 | 0 |
| k__Eukaryota;p__Bacillariophyta;c__Bacillariophyceae;o__Bacillariales;f__Bacillariaceae;g__Bacillaria | 0 | 0 | 0 | 2 | 30 | 0 | 0 | 0 |
| k__Eukaryota;p__unidentified;c__Dinophyceae;o__Gonyaulacales;f__Gonyaulacaceae;g__Alexandrium | 3 | 5 | 0 | 0 | 4 | 15 | 0 | 5 |
| k__Eukaryota;p__unidentified;c__unidentified;o__Cercomonadida;f__Cercomonadidae;g__Cercomonas | 0 | 0 | 0 | 23 | 7 | 0 | 0 | 0 |
| k__Eukaryota;p__Bacillariophyta;c__Coscinodiscophyceae;o__Rhizosoleniales;f__Rhizosoleniaceae;g__Proboscia | 29 | 0 | 0 | 0 | 0 | 0 | 0 | 0 |
| k__Eukaryota;p__Bacillariophyta;c__Mediophyceae;o__Cymatosirales;f__Cymatosiraceae;g__Cymatosira | 0 | 0 | 0 | 22 | 7 | 0 | 0 | 0 |
| k__Eukaryota;p__unidentified;c__unidentified;o__Pyrenomonadales;f__Geminigeraceae;g__Proteomonas | 0 | 0 | 0 | 0 | 0 | 0 | 8 | 20 |
| k__Eukaryota;p__unidentified;c__unidentified;o__unidentified;f__Acanthoecidae;g__Acanthoeca | 8 | 0 | 0 | 0 | 0 | 0 | 20 | 0 |
| k__Eukaryota;p__Bacillariophyta;c__Mediophyceae;o__Hemiaulales;f__Hemiaulaceae;g__Cerataulina | 0 | 0 | 0 | 0 | 4 | 0 | 0 | 23 |
| k__Eukaryota;p__Bacillariophyta;c__Mediophyceae;o__Cymatosirales;f__Cymatosiraceae;g__Minutocellus | 0 | 19 | 2 | 0 | 5 | 0 | 0 | 0 |
| k__Eukaryota;p__Chlorophyta;c__unidentified;o__Pyramimonadales;f__unidentified;g__Pterosperma | 25 | 0 | 0 | 0 | 0 | 0 | 0 | 0 |
| k__Eukaryota;p__Bacillariophyta;c__Bacillariophyceae;o__Bacillariales;f__Bacillariaceae;g__Cylindrotheca | 0 | 15 | 0 | 0 | 0 | 0 | 0 | 9 |
| k__Eukaryota;p__unidentified;c__Dinophyceae;o__Syndiniales;f__Syndiniaceae;g__Syndinium | 0 | 7 | 0 | 17 | 0 | 0 | 0 | 0 |
| k__Eukaryota;p__unidentified;c__unidentified;o__unidentified;f__Stephanoecidae;g__Diaphanoeca | 3 | 0 | 0 | 0 | 14 | 0 | 0 | 7 |
| k__Eukaryota;p__Bacillariophyta;c__Bacillariophyceae;o__Naviculales;f__Naviculaceae;g__Haslea | 23 | 0 | 0 | 0 | 0 | 0 | 0 | 0 |
| k__Eukaryota;p__unidentified;c__unidentified;o__unidentified;f__Salpingoecidae;g__Lagenoeca | 0 | 0 | 0 | 2 | 12 | 0 | 0 | 9 |
| k__Eukaryota;p__unidentified;c__Dinophyceae;o__Dinophysiales;f__Dinophysiaceae;g__Phalacroma | 8 | 0 | 0 | 0 | 8 | 0 | 0 | 6 |
| k__Eukaryota;p__Bacillariophyta;c__Bacillariophyceae;o__unidentified;f__unidentified;g__unidentified | 0 | 21 | 0 | 0 | 0 | 0 | 0 | 0 |
| k__Eukaryota;p__unidentified;c__Dinophyceae;o__Gymnodiniales;f__Gymnodiniaceae;g__Apicoporus | 1 | 0 | 0 | 10 | 3 | 3 | 0 | 4 |
| k__Eukaryota;p__unidentified;c__Dinophyceae;o__Peridiniales;f__Heterocapsaceae;g__Cachonina | 1 | 2 | 0 | 0 | 0 | 1 | 0 | 16 |
| k__Eukaryota;p__unidentified;c__unidentified;o__unidentified;f__unidentified;g__Chrysoculter | 20 | 0 | 0 | 0 | 0 | 0 | 0 | 0 |
| k__Eukaryota;p__unidentified;c__Dinophyceae;o__Peridiniales;f__Diplopsaliaceae;g__Diplopsalis | 2 | 0 | 1 | 0 | 0 | 0 | 5 | 11 |
| k__Eukaryota;p__Bacillariophyta;c__Coscinodiscophyceae;o__Thalassiosirales;f__Stephanodiscaceae;g__Cyclotella | 15 | 0 | 0 | 0 | 3 | 0 | 0 | 0 |
| k__Eukaryota;p__unidentified;c__Chrysophyceae;o__Chromulinales;f__Chrysolepidomonadaceae;g__Chrysolepidomonas | 0 | 0 | 0 | 0 | 17 | 0 | 0 | 0 |
| k__Eukaryota;p__unidentified;c__unidentified;o__unidentified;f__Acanthoecidae;g__Pleurasiga | 0 | 0 | 0 | 0 | 6 | 11 | 0 | 0 |
| k__Eukaryota;p__Bacillariophyta;c__Coscinodiscophyceae;o__Thalassiosirales;f__Skeletonemataceae;g__Skeletonema | 0 | 0 | 0 | 14 | 2 | 0 | 0 | 0 |
| k__Eukaryota;p__unidentified;c__Dinophyceae;o__Gonyaulacales;f__Calciodinellaceae;g__Posoniella | 6 | 0 | 0 | 0 | 0 | 0 | 0 | 10 |
| k__Eukaryota;p__unidentified;c__Dinophyceae;o__Peridiniales;f__Peridiniaceae;g__Durinskia | 1 | 0 | 0 | 7 | 5 | 3 | 0 | 0 |
| k__Eukaryota;p__unidentified;c__Dinophyceae;o__Gymnodiniales;f__Gymnodiniaceae;g__Paragymnodinium | 15 | 0 | 0 | 0 | 0 | 0 | 0 | 0 |
| k__Eukaryota;p__unidentified;c__unidentified;o__Euglyphida;f__Paulinellidae;g__Paulinella | 0 | 6 | 0 | 0 | 9 | 0 | 0 | 0 |
| k__Eukaryota;p__unidentified;c__unidentified;o__unidentified;f__unidentified;g__Pseudopirsonia | 0 | 0 | 0 | 6 | 0 | 0 | 0 | 9 |
| k__Eukaryota;p__Chlorophyta;c__Chlorodendrophyceae;o__Chlorodendrales;f__Chlorodendraceae;g__Tetraselmis | 0 | 0 | 0 | 0 | 0 | 0 | 0 | 14 |
| k__Eukaryota;p__unidentified;c__Dinophyceae;o__Peridiniales;f__Amphidiniopsidaceae;g__Archaeperidinium | 0 | 0 | 0 | 14 | 0 | 0 | 0 | 0 |
| k__Eukaryota;p__unidentified;c__Dinophyceae;o__Prorocentrales;f__Prorocentraceae;g__Plagiodinium | 6 | 0 | 0 | 0 | 8 | 0 | 0 | 0 |
| k__Eukaryota;p__unidentified;c__unidentified;o__Glissomonadida;f__Viridiraptoridae;g__Viridiraptor | 8 | 0 | 0 | 6 | 0 | 0 | 0 | 0 |
| k__Eukaryota;p__Chlorophyta;c__Trebouxiophyceae;o__Microthamniales;f__unidentified;g__Trebouxia | 13 | 0 | 0 | 0 | 0 | 0 | 0 | 0 |
| k__Eukaryota;p__Bacillariophyta;c__Fragilariophyceae;o__Thalassionemales;f__Thalassionemataceae;g__Thalassionema | 4 | 0 | 0 | 0 | 0 | 0 | 0 | 8 |
| k__Eukaryota;p__Chlorophyta;c__Mamiellophyceae;o__Dolichomastigales;f__Dolichomastigaceae;g__Dolichomastix | 0 | 0 | 0 | 0 | 0 | 0 | 0 | 12 |
| k__Eukaryota;p__unidentified;c__Synurophyceae;o__Synurales;f__Mallomonadaceae;g__Mallomonas | 10 | 0 | 0 | 0 | 0 | 2 | 0 | 0 |
| k__Eukaryota;p__Chlorophyta;c__Mamiellophyceae;o__Dolichomastigales;f__Crustomastigaceae;g__Crustomastix | 3 | 0 | 0 | 0 | 5 | 3 | 0 | 0 |
| k__Eukaryota;p__unidentified;c__unidentified;o__Cryptomonadales;f__Cryptomonadaceae;g__Cryptomonas | 0 | 0 | 11 | 0 | 0 | 0 | 0 | 0 |
| k__Eukaryota;p__unidentified;c__unidentified;o__Cryptomonadales;f__Goniomonadaceae;g__Goniomonas | 3 | 0 | 0 | 2 | 0 | 0 | 0 | 6 |
| k__Eukaryota;p__unidentified;c__Synurophyceae;o__Parmales;f__Triparmaceae;g__Triparma | 0 | 0 | 6 | 0 | 4 | 0 | 0 | 0 |
| k__Eukaryota;p__unidentified;c__unidentified;o__Cryptomonadales;f__Hemiselmidaceae;g__Hemiselmis | 0 | 1 | 0 | 0 | 0 | 0 | 0 | 9 |
| k__Eukaryota;p__unidentified;c__unidentified;o__Ichthyophonida;f__Amoebidiaceae;g__Amoebidium | 0 | 7 | 0 | 3 | 0 | 0 | 0 | 0 |
| k__Eukaryota;p__unidentified;c__unidentified;o__Zygodiscales;f__Helicosphaeraceae;g__Helicosphaera | 3 | 4 | 1 | 0 | 0 | 2 | 0 | 0 |
| k__Eukaryota;p__unidentified;c__unidentified;o__Phaeogromida;f__Challengeriidae;g__Protocystis | 9 | 0 | 0 | 0 | 0 | 0 | 0 | 0 |
| k__Eukaryota;p__Xanthophyceae;c__unidentified;o__Mischococcales;f__Botryidiopsidaceae;g__Botrydiopsis | 9 | 0 | 0 | 0 | 0 | 0 | 0 | 0 |
| k__Eukaryota;p__Bacillariophyta;c__Mediophyceae;o__Cymatosirales;f__Cymatosiraceae;g__Pseudoleyanella | 5 | 0 | 0 | 1 | 0 | 0 | 0 | 2 |
| k__Eukaryota;p__Chlorophyta;c__Mamiellophyceae;o__Mamiellales;f__Bathycoccaceae;g__Bathycoccus | 0 | 0 | 0 | 0 | 0 | 5 | 3 | 0 |
| k__Eukaryota;p__Bacillariophyta;c__Coscinodiscophyceae;o__Thalassiosirales;f__Thalassiosiraceae;g__Porosira | 0 | 0 | 0 | 7 | 0 | 0 | 0 | 0 |
| k__Eukaryota;p__unidentified;c__Chrysophyceae;o__Chromulinales;f__Chromulinaceae;g__Pedospumella | 7 | 0 | 0 | 0 | 0 | 0 | 0 | 0 |
| k__Eukaryota;p__unidentified;c__unidentified;o__unidentified;f__Stephanoecidae;g__Calliacantha | 0 | 0 | 0 | 0 | 7 | 0 | 0 | 0 |
| k__Eukaryota;p__unidentified;c__unidentified;o__unidentified;f__unidentified;g__Lotharella | 2 | 0 | 0 | 0 | 0 | 0 | 0 | 5 |
| k__Eukaryota;p__Bacillariophyta;c__Bacillariophyceae;o__Naviculales;f__Naviculaceae;g__Navicula | 0 | 0 | 0 | 0 | 6 | 0 | 0 | 0 |
| k__Eukaryota;p__Chlorophyta;c__Trebouxiophyceae;o__Chlorellales;f__Leptosiraceae;g__Pseudopleurococcus | 6 | 0 | 0 | 0 | 0 | 0 | 0 | 0 |
| k__Eukaryota;p__Chlorophyta;c__Ulvophyceae;o__Ulotrichales;f__Chlorocystidaceae;g__Halochlorococcum | 0 | 0 | 0 | 0 | 0 | 0 | 0 | 6 |
| k__Eukaryota;p__unidentified;c__Dictyochophyceae;o__Pedinellales;f__unidentified;g__Pteridomonas | 0 | 0 | 0 | 0 | 6 | 0 | 0 | 0 |
| k__Eukaryota;p__unidentified;c__Dinophyceae;o__Dinophysiales;f__Dinophysiaceae;g__Dinophysis | 0 | 0 | 0 | 0 | 2 | 0 | 0 | 3 |
| k__Eukaryota;p__unidentified;c__Dinophyceae;o__unidentified;f__unidentified;g__Balechina | 0 | 0 | 0 | 3 | 0 | 0 | 0 | 2 |
| k__Eukaryota;p__Bacillariophyta;c__Bacillariophyceae;o__Bacillariales;f__Bacillariaceae;g__Fragilariopsis | 4 | 0 | 0 | 0 | 0 | 0 | 0 | 0 |
| k__Eukaryota;p__Bacillariophyta;c__Bacillariophyceae;o__Naviculales;f__Pleurosigmataceae;g__Pleurosigma | 0 | 0 | 0 | 0 | 4 | 0 | 0 | 0 |
| k__Eukaryota;p__Bacillariophyta;c__Mediophyceae;o__Triceratiales;f__Plagiogrammaceae;g__Dimeregramma | 4 | 0 | 0 | 0 | 0 | 0 | 0 | 0 |
| k__Eukaryota;p__Chlorophyta;c__unidentified;o__Prasinococcales;f__unidentified;g__Prasinoderma | 2 | 0 | 0 | 0 | 0 | 0 | 0 | 2 |
| k__Eukaryota;p__unidentified;c__Chrysophyceae;o__Chromulinales;f__Dinobryaceae;g__Dinobryon | 0 | 0 | 0 | 0 | 4 | 0 | 0 | 0 |
| k__Eukaryota;p__unidentified;c__Dinophyceae;o__Pyrocystales;f__Pyrocystaceae;g__Pyrocystis | 4 | 0 | 0 | 0 | 0 | 0 | 0 | 0 |
| k__Eukaryota;p__unidentified;c__unidentified;o__Cryomonadida;f__unidentified;g__Ventrifissura | 0 | 0 | 0 | 0 | 4 | 0 | 0 | 0 |
| k__Eukaryota;p__unidentified;c__unidentified;o__unidentified;f__Salpingoecidae;g__Monosiga | 4 | 0 | 0 | 0 | 0 | 0 | 0 | 0 |
| k__Eukaryota;p__unidentified;c__unidentified;o__unidentified;f__Salpingoecidae;g__Salpingoeca | 0 | 0 | 2 | 0 | 0 | 2 | 0 | 0 |
| k__Eukaryota;p__Bacillariophyta;c__Coscinodiscophyceae;o__Coscinodiscales;f__Coscinodiscaceae;g__Palmerina | 2 | 1 | 0 | 0 | 0 | 0 | 0 | 0 |
| k__Eukaryota;p__Bacillariophyta;c__Mediophyceae;o__Cymatosirales;f__Cymatosiraceae;g__Brockmanniella | 3 | 0 | 0 | 0 | 0 | 0 | 0 | 0 |
| k__Eukaryota;p__Bolidophyceae;c__unidentified;o__unidentified;f__unidentified;g__Bolidomonas | 0 | 0 | 3 | 0 | 0 | 0 | 0 | 0 |
| k__Eukaryota;p__Chlorophyta;c__Ulvophyceae;o__Ulvales;f__Ulvaceae;g__Ulva | 0 | 0 | 0 | 0 | 0 | 0 | 3 | 0 |
| k__Eukaryota;p__unidentified;c__Dinophyceae;o__Noctilucales;f__Kofoidiniaceae;g__Kofoidinium | 0 | 0 | 0 | 3 | 0 | 0 | 0 | 0 |
| k__Eukaryota;p__unidentified;c__unidentified;o__unidentified;f__Ellobiopsidae;g__Ellobiopsis | 0 | 2 | 0 | 0 | 0 | 0 | 0 | 0 |
| k__Eukaryota;p__unidentified;c__unidentified;o__unidentified;f__Planomonadidae;g__Planomonas | 0 | 0 | 0 | 0 | 0 | 2 | 0 | 0 |
| k__Eukaryota;p__unidentified;c__unidentified;o__unidentified;f__unidentified;g__Solenicola | 2 | 0 | 0 | 0 | 0 | 0 | 0 | 0 |
